# Prediction of sepsis mortality using metabolite biomarkers in the blood: a meta-analysis of death-related pathways and prospective validation

**DOI:** 10.1186/s12916-020-01546-5

**Published:** 2020-04-15

**Authors:** Jing Wang, Yizhu Sun, Shengnan Teng, Kefeng Li

**Affiliations:** 1 Department of Critical Care Medicine, Yantai Yuhuangding Hospital, Yantai, 264000 Shandong China; 2 School of Medicine, University of California, San Diego, CA 92103 USA

**Keywords:** Sepsis, Metabolomics, Blood, Prediction, Outcome, Death-related metabolic pathways, Meta-analysis

## Abstract

**Background:**

Sepsis is a leading cause of death in intensive care units (ICUs), but outcomes of individual patients are difficult to predict. The recently developed clinical metabolomics has been recognized as a promising tool in the clinical practice of critical illness. The objective of this study was to identify the unique metabolic biomarkers and their pathways in the blood of sepsis nonsurvivors and to assess the prognostic value of these pathways.

**Methods:**

We searched PubMed, EMBASE, Cochrane, Web of Science, CNKI, Wangfang Data, and CQVIP from inception until July 2019. Eligible studies included the metabolomic analysis of blood samples from sepsis patients with the outcome. The metabolic pathway was assigned to each metabolite biomarker. The meta-analysis was performed using the pooled fold changes, area under the receiver operating characteristic curve (AUROC), and vote-counting of metabolic pathways. We also conducted a prospective cohort metabolomic study to validate the findings of our meta-analysis.

**Results:**

The meta-analysis included 21 cohorts reported in 16 studies with 2509 metabolite comparisons in the blood of 1287 individuals. We found highly limited overlap of the reported metabolite biomarkers across studies. However, these metabolites were enriched in several death-related metabolic pathways (DRMPs) including amino acids, mitochondrial metabolism, eicosanoids, and lysophospholipids. Prediction of sepsis death using DRMPs yielded a pooled AUROC of 0.81 (95% CI 0.76–0.87), which was similar to the combined metabolite biomarkers with a merged AUROC of 0.82 (95% CI 0.78–0.86) (*P* > 0.05). A prospective metabolomic analysis of 188 sepsis patients (134 survivors and 54 nonsurvivors) using the metabolites from DRMPs produced an AUROC of 0.88 (95% CI 0.78–0.97). The sensitivity and specificity for the prediction of sepsis death were 80.4% (95% CI 66.9–89.4%) and 78.8% (95% CI 62.3–89.3%), respectively.

**Conclusions:**

DRMP analysis minimizes the discrepancies of results obtained from different metabolomic methods and is more practical than blood metabolite biomarkers for sepsis mortality prediction.

**Trial registration:**

The meta-analysis was registered on OSF Registries, and the prospective cohort study was registered on the Chinese Clinical Trial Registry (ChiCTR1800015321).

## Background

Sepsis is defined as the potentially life-threatening condition caused by the body’s extreme response to infection. Sepsis is one of the leading causes of death worldwide, and nearly 6 million people die of sepsis all over the world annually [1, 2]. Despite the increasing use of advanced technology for its treatment, such as bundled early goal-directed therapy (EGDT) [3], the prognosis of sepsis remains poor. The high mortality rates of sepsis are partially due to the lack of an effective approach to predict sepsis outcomes. In addition, only a small number of studies had investigated the molecular mechanisms of sepsis-induced organ failure and death [4, 5].

Several traditional sepsis outcome prediction approaches are currently used in clinical practice, such as the Sequential Organ Failure Assessment (SOFA) [6], the Acute Physiology and Chronic Health Evaluation II (APACHE II) [7], and the Simplified Acute Physiology Score II (SAPS II) [8]. However, their performance (sensitivity, specificity) has not been shown to be adequate for all cases [9]. There is a critical need to understand the mechanisms of sepsis-induced death and to identify better prognostic models that would facilitate the development of adapted strategies for different cases of sepsis.

The recently developed omics techniques facilitate high-throughput screening of disease-specific biomarkers in biological fluids, of which metabolomics is one of the most promising approaches [10]. Metabolomics aims to measure the small molecules (metabolites) within cells, biofluids, tissues, or organisms using various analytical techniques. Unlike genomics, transcriptomics, and proteomics, metabolomics represents the molecular phenotype of an organism because metabolites and their concentrations are the direct functional “readout” of cellular activity and the state of cells and tissues [11]. Therefore, clinical metabolomics offers a strategic advantage for the elucidation of the new roles of metabolism in disease, the identification of biomarkers, and the development of new therapeutics [11–16].

Recent studies have highlighted the potential prognostic role of metabolomics in sepsis patients [17–19]. Although promising, the existing literature of metabolomic studies on sepsis mortality prediction is limited by the use of individual cohorts with small sample sizes and low statistical power, as well as the use of varying analytical pipelines that can make it challenging to synthesize findings. Furthermore, the lack of validation in independent cohorts limits the clinical utility of metabolomic profiling in sepsis outcome prediction [20–22]. Meta-analysis can overcome these limitations by increasing the sample size and thus statistical power to generate the best estimation. Unfortunately, no studies have performed either descriptive or quantitative meta-analysis of metabolomics for predicting sepsis outcomes.

In this study, we systematically reviewed the literature to identify all eligible clinical metabolomic studies containing the prognosis of sepsis published before July 2019. We retrospectively generated a comprehensive dataset and performed both descriptive and quantitative meta-analysis using the curated dataset. In addition, we conducted a prospective metabolomic cohort study to validate the findings in the meta-analysis. The primary goals of this study were to identify the metabolic biomarkers and their pathways in the blood of sepsis nonsurvivors and to assess the prognostic significance of metabolomic profiling in sepsis patients.

## Methods

### Search strategy

We performed a comprehensive literature search of articles through the following databases without date limitation: PubMed, EMBASE, the Cochrane Library, Web of Science, China National Knowledge Infrastructure (CNKI), Wanfangdata, and CQVIP. The search was updated to July 1, 2019, and not restricted by language. The main search terms included the following: “Sepsis” (e.g., “Severe Sepsis” and “Sepsis, Severe” and “Pyemia” and “Pyemias” and “Pyohemia” and “Pyohemias” and “Pyaemia” and “Pyaemias” and “Septicemia” and “Septicemias” and “Poisoning, Blood” and “Blood Poisoning” and “Poisonings, Blood” and “Bacteremia” and “Endotoxemia” and “Fungemia” and “Candidemia” and “Parasitemia” and “Viremia”) and “Metabolomics” (e.g., “Metabolomic” and “Metabonomics” and “Metabonomic”). The reference list was also checked for relevant articles. The detailed search strategy is listed in Additional file 1: Supplemental methods.

### Inclusion and exclusion criteria

The inclusion criteria for selecting the studies for this meta-analysis were as follows: (1) metabolomic profiling performed in patients with sepsis or septic shock according to the published diagnosis criteria [23], (2) metabolites were measured in plasma or serum, (3) sepsis survivors versus sepsis nonsurvivors, (4) mortality data and the correlation of metabolites with sepsis survivors and/or nonsurvivors were reported, and (5) the type of study design was not restricted. The exclusion criteria were as follows: (1) patients < 18 years old and studies involving animals, (2) studies without the sepsis outcome, and (3) meeting abstracts, letters to the editor, case reports, and reviews.

### Data extraction and quality assessment

All potentially eligible articles were independently evaluated, and the information was extracted by two authors (JW and ST). Disagreements were resolved by discussion with a third person (YS). For each study, the following items were extracted: first author, year of publication, country, type of patients, total number of cases and gender, follow-up time, mortality, clinical scores (i.e., SOFA and APACHE II), the analytical platforms, metabolites with significant changes, metabolite fold change (FC, nonsurvivors/survivor), adjusted *P* value, area under receiver operating characteristic (ROC) curve (AUROC), and validation. Because of the inconsistency of the names for the same reported metabolites between publications, especially for the nomenclature of lipids, we then used the software OpenRefine (https://openrefine.org/) and the ID conversion tool in MetaboAnalyst 4.0 (www.metaboanalyst.ca) to match the names in the publications to the names in HMDB or PubChem. We removed the ambiguity by adding the identifiers to each metabolite if available (CAS, HMDB, or KEGG). We performed cross-data quality checks between reviewers at each step and reviewed all the included references after dataset construction.

Newcastle-Ottawa Scale (NOS) was used to assess the risk of bias for each of the included studies by two authors independently. The NOS has three parts: selection (0–4 points), comparability (0–2 points), and outcome assessment (0–3 points). We made slight modifications in the parts of selection and outcome assessment in original NOS based on the guidelines for a reliable metabolomic study [24, 25]. Both original and modified NOS scores are reported (Additional file 1: Table S1 and Table S2). Details for NOS and modified NOS are available in Additional file 1: Supplemental methods.

### Data synthesis and meta-analysis

For pathway analysis, we assigned both the chemical class and the biochemical pathway to each metabolite according to HMDB and our in-house database. Cochran’s *Q* test and Higgins *I*-squared statistic were used to assess the heterogeneity across the included studies. The pooled AUROC and FC were calculated using a random model when the heterogeneity was statistically significant across the studies (*P* < 0.10 or *I*^2^ > 50%). Otherwise, a fixed-effect model was used. Sensitivity analyses were conducted by serially excluding each study to determine the influence of individual studies on the pooled AUROC. Publication bias was evaluated using funnel plots. The meta-analysis was performed using MedCalc 19.0.7. The frequency of a particular pathway (chemical class) reported to be significant across the studies was analyzed by the vote-counting method.

### Validation of meta-analysis results using a prospective cohort metabolomic study

This prospective cohort study was approved by the Institutional Review Board (IRB) of Yantai Yuhuangding Hospital ([2018]11) and registered on the Chinese Clinical Trial Registry (Registry ID: ChiCTR1800015321). All the protocols conformed to the World Medical Association Declaration of Helsinki-Ethical Principles for Medical Research Involving Human Subjects. Briefly, the diagnostic evaluation was performed upon admission to the intensive care unit (ICU) of Yantai Yuhuangding Hospital, and patients who met the Third International Consensus Definitions for Sepsis and Septic Shock were eligible for selection [26]. The exclusion criteria included the following: (1) younger than 18 years or older than 85 years of age, (2) diabetes and other metabolic-related diseases, (3) AIDS, (4) pregnant women, and (5) incomplete clinical data.

A total of 188 patients were enrolled between June 2017 and May 2018, and written permission was obtained from all the patients or their guardians. SOFA and APACHE II scores were assessed during the first 24 h of ICU admission. Heparinized plasma samples were collected at the time of ICU admission and stored at − 80 °C until analysis. The patients were followed up for the survival status weekly for 28 days. On day 28, out of 188 patients enrolled, 134 survived and 54 died. We then divided the plasma samples collected during admission into two groups: survivors (*N* = 134) and nonsurvivors (*N* = 54).

Metabolomic analysis was performed on extracted metabolites as described previously [27–29]. The chromatographic peaks were identified using MultiQuant (v3.0, AB SCIEX), confirmed by manual inspection, and the peak areas were integrated. The data were log2 transformed before statistical analysis. Partial least squares discriminant analysis (PLS-DA) was conducted in MetaboAnalyst 4.0. Seven metabolites including isoleucine (amino acids), alanine (amino acids), acetylcarnitine (mitochondrial metabolism), lactic acid (mitochondrial metabolism), pyruvic acid (mitochondrial metabolism), LysoPG (22:0) (lysophospholipid metabolism), and LysoPC (24:0) (lysophospholipid metabolism) were selected based on the results of the meta-analysis. Multivariate ROC analysis was conducted using MetaboAnalyst 4.0 (https://www.metaboanalyst.ca). The ROC curve was generated based on Monte Carlo cross-validation of random forest models [30]. Repeated random cross-validation (rdCV) and permutation test were used for internal validation of the established classification model.

Other statistical analyses were conducted in GraphPad Prism 8.0. All statistical tests were 2-tailed, and the significance threshold (*P*) was set at 0.05.

## Results

### Study inclusion and characteristics

**Fig. 1:**
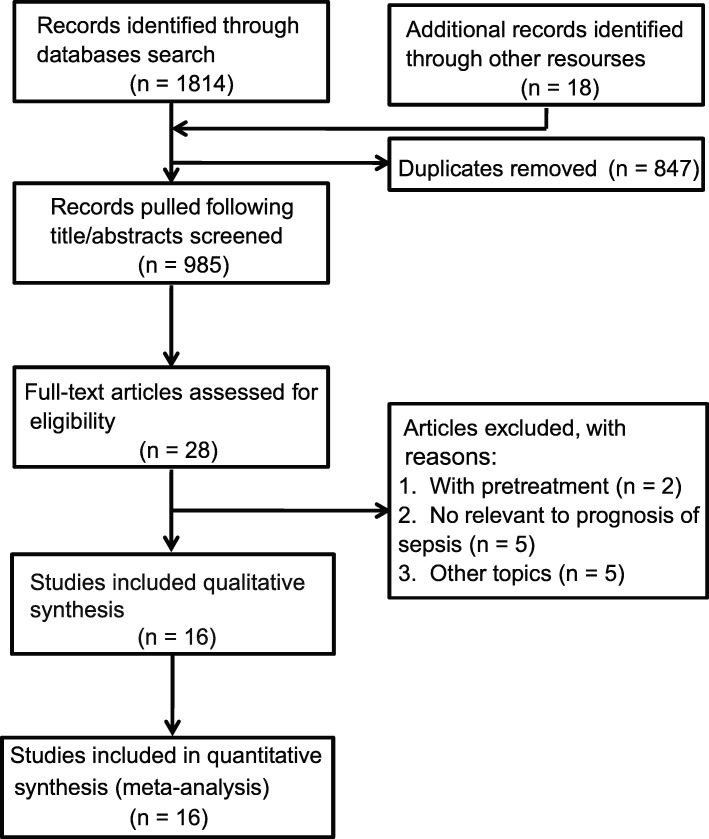
Flow chart of the included studies

The initial search strategies retrieved a total of 1814 articles. After meticulous inspection of the articles, 16 clinical metabolomic studies published between 2003 and 2019 were finally enrolled in our meta-analysis, in which sepsis survivors were compared to the sepsis nonsurvival group (Additional File 1: Table S3) [31–46]. The processes of study selection are summarized in a flow diagram (Fig. 1).

Studies related to sepsis mortality prediction using metabolomic approaches were mostly published after 2013, underscoring the emerging nature of this field of research. Among them, 7 studies were from the USA; 3 studies were performed in China; 2 studies were conducted in France and Canada, respectively; and 1 in Germany and Italy, respectively (Additional File 1: Table S3). APACHE and SOFA scores for mortality prediction were both reported in 7 studies. One study had both SOFA and SAPS scores, 2 studies reported SOFA scores, and 4 studies reported APACHE scores. Traditional prognostic scores were missing in 2 studies.

Sepsis is a severe critical illness syndrome with multi-organ dysfunction, and tissue biopsy is generally not performed for patients with sepsis. In addition, the urine metabolome shows more interindividual and technical variability than that of the blood [47]. Therefore, in our meta-analysis, we chose only blood-based (plasma and serum) metabolomic studies (10 studies with plasma and 6 studies with serum) (Table 1). Overall, the resulting collection contains curated quality-checked data of 21 cohorts reported in 16 studies and over 2509 metabolite comparisons in blood from 1287 individuals (Additional file 1: Table S4).

**Table 1:** The characteristics of the included studies

PubMed ID	Patient no. (M/F)	Comparison groups (S vs NS)	Age (years, median range, or SD) (S vs NS)	Follow-up	SOFA (S vs NS)	APACHE II (S vs NS)	SAPS II (S vs NS)	Matrix
PMID12562829	102 (71/31)	S (*n* = 63) vs NS (*n* = 39)	53.8 (20–91) vs 54.9 (17–80)	30 days	N/A	N/A	N/A	Plasma
PMID23673400	30 (16/14)	S (*n* = 15) vs NS (*n* = 15)	78 (73–83) vs 79 (76–82)	90 days	N/A	59 (53–75) vs 76 (74–95)^#^	N/A	Plasma
PMID23884467	121 70/51)	S (*n* = 90) vs NS (*n* = 31)	56.4 ± 19.2 vs 68.8 ± 16.7	28 days	4.3 ± 2.7 vs 7.0 ± 3.6	15.0 ± 7.1 vs 22.8 ± 7.8	N/A	Plasma
PMID23884467	52* (34/18)	S (*n* = 34) vs NS (*n* = 18)	58.9 ± 18.1 vs 58.0 ± 18.8	28 days	4.3 ± 2.7 vs 5.0 ± 3.0	5.7 ± 5.8 vs 18.5 ± 7.9	N/A	Plasma
PMID23884467	61* (33/28)	S (*n* = 36) vs NS (*n* = 25)	54.8 ± 13.1 vs 58.7 ± 16.5	28 days	N/A	25.9 ± 6.7 vs 33.0 ± 9.8	N/A	Plasma
PMID24368342	8	S (*n* = 4) vs NS (*n* = 4)	61 (56–70)	N/A	11 (5–14)	22 (16–27)	N/A	Serum
PMID24498130	90 (39/51)	S (*n* = 60) vs NS (*n* = 30)	53 ± 14 vs 58 ± 15	28 days	N/A	23 ± 9 vs 30 ± 11	N/A	Plasma
PMID24498130	149* 68/81)	S (*n* = 115) vs NS (*n* = 34)	58 ± 17 vs 69 ± 16	28 days	N/A	15 ± 7 vs 23 ± 8	N/A	Plasma
PMID25553245	35 (30/5)	S (*n* = 26) vs NS (*n* = 9)	63 ± 18 vs 67 ± 15	48 h	10 ± 4 vs 13 ± 5	20 ± 8 vs 26 ± 6	N/A	Serum
PMID25849571	35 (25/10)	S (*n* = 20) vs NS (*n* = 15)	54 ± 23 vs 61 ± 21	28 days	7 ± 4 vs 10 ± 5	14 ± 7 vs 22 ± 8	N/A	Serum
PMID25887472	121 (70/51)	S (*n* = 90) vs NS (*n* = 31)	56.4 ± 19.2 vs 68.8 ± 16.7	28 days	4.3 ± 2.7 vs 7.0 ± 3.6	15.0 ± 7.1 vs 22.8 ± 7.8	N/A	Plasma
PMID25928796	16	S (*n* = 8) vs NS (*n* = 8)	63 (59.8–77)	N/A	10.5 (7–12.5)	25.5 (17.5–31.3)	N/A	Serum
PMID26847922	20 (13/7)	S (*n* = 9) vs NS (*n* = 11)	61.3 ± 15.2 vs 69.9 ± 12	90 days	10.5 ± 1.5 vs 12.1 ± 2	N/A	54.8 ± 17.9 vs 61.1 ± 9.4	Plasma
PMID27406941	58	S (*n* = 28) vs NS (*n* = 30)	N/A	28 days	N/A	N/A	N/A	Plasma
PMID27614981	50 (27/23)	S (*n* = 21) vs NS (*n* = 29)	63.8 ± 0.7 vs 65.6 ± 0.5	7 days	10.0 ± 0.8 vs 11.3 ± 0.9	N/A	54 ± 0.4 vs 68 ± 0.5	Serum
PMID27632672	22 (13/9)	S (*n* = 13) vs NS (*n* = 9)	60 (27–84) vs 60 (36–80)	7 days	N/A	22 (14–38) vs 31 (16–46)	N/A	Plasma
PMID28345042	36 (27/9)	S (*n* = 20) vs NS (*n* = 16)	52 ± 21.5 vs 58 ± 16.7	28 days	N/A	17 ± 4.9 vs 22 ± 10.2	N/A	Plasma
PMID28345042	121* (70/51)	S (*n* = 90) vs NS (*n* = 31)	56.4 ± 19.2 vs 68.8 ± 16.7	28 days	4.3 ± 2.7 vs 7.0 ± 3.6	15.0 ± 7.1 vs 22.8 ± 7.8	N/A	Plasma
PMID30379669	90 (54/36)	S (*n* = 69) vs NS (*n* = 21)	71.5 ± 15.4 vs 69.6 ± 12.9	28 days	8.3 ± 3.5 vs 10.9 ± 4.0	23.1 ± 7.7 vs 26.1 ± 8.6	N/A	Plasma
PMID31088568	70 (40/30)	S (*n* = 40) vs NS (*n* = 30)	68.5 ± 0.3 vs 72.1 ± 0.4	24 h	10.9 ± 0.1 vs 12.4 ± 0.1	N/A	N/A	Serum
PMID31088568	70* (40/30)	S (*n* = 40) vs NS (*n* = 30)	68.5 ± 0.3 vs 72.1 ± 0.4	24 h	10.9 ± 0.1 vs 12.4 ± 0.1	N/A	55.2 ± 0.4 vs 64.3 ± 0.6	Serum

*Abbreviations*: *S* survivors, *NS* nonsurvivors, *SOFA* The Sequential Organ Failure Assessment, *APACHE II* Acute Physiology and Chronic Health Evaluation II, *SAPS II* Simplified Acute Physiology Score, *N/A* not available

*Validation cohort in the same publication

^#^APACHE III

### Quality assessment of clinical metabolomic studies

Good quality control and proper validation are the prerequisites for the success of a clinical metabolomic study [48]. The pooled QC samples were used in all MS-driven metabolomic studies of the prognosis of sepsis. Only 6 out of 16 studies reported validations either by animal studies, independent cohorts, or other omics techniques (Additional file 1: Table S5). Therefore, this meta-analysis is useful for validating the findings of individual studies. The details of quality assessment are described in Additional file 1: Supplemental results.

### Meta-analysis of chemical classes and biochemical pathways using the vote-counting method

We first checked the overlap in metabolite biomarkers used for prediction of sepsis mortality across the studies but found few in common (Additional file 1: Figure S1). We then explored the unique metabolic features in sepsis nonsurvivors compared to the survivors by analyzing the chemical classes and metabolic pathways of the biomarkers.

#### Chemical classes

**Fig. 2:**
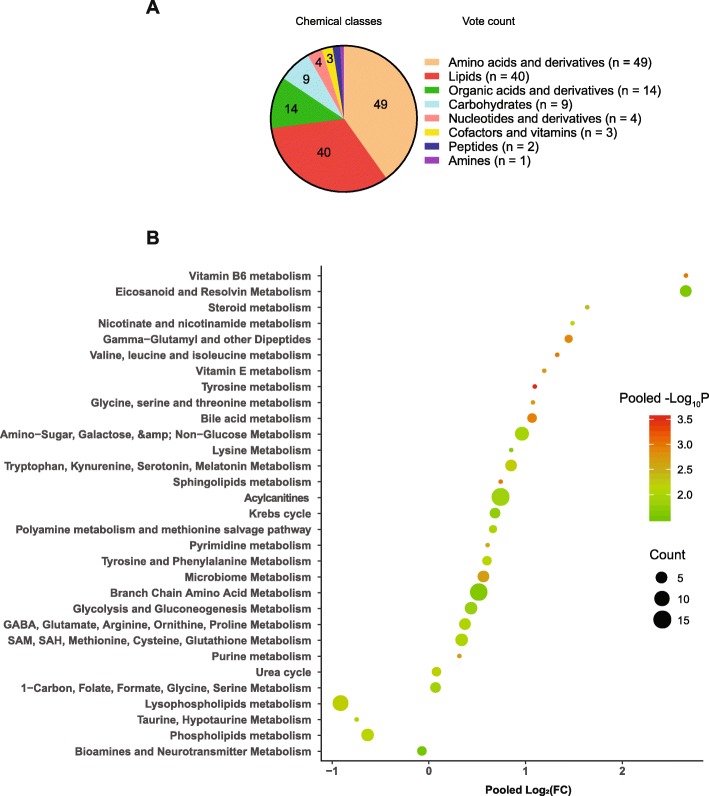
The vote count of the chemical classes for the differential metabolites between survivors and nonsurvivors (**a**) and the pooled fold changes (nonsurvivors/survivors), and *P* values for the dramatically altered metabolic pathways in sepsis nonsurvivors compared to the survivors (**b**). For the pie chart (**a**), the vote count indicated the frequency of a chemical class being identified as statistically different between sepsis nonsurvivors and survivors. For the volcano plot (**b**), a metabolic pathway was assigned to each differential metabolite. The pooled fold change and *P* value were calculated using random effects in the meta-analysis. We also added the vote-counting analysis to the volcano plot, which showed the frequency of a metabolic pathway being identified as statistically different between sepsis nonsurvivors and survivors. *P* < 0.05 was considered statistically significant

We assigned the chemical class to each of the biomarkers based on the classification in HMDB (Additional file 1: Table S6). The main chemical classes for metabolites with prognostic values for sepsis are amino acid and derivatives, lipids and lipid-like molecules, and organic acids and derivatives (Fig. 2a).

#### Metabolic pathways

We next analyzed the metabolic pathways for the differential metabolites between sepsis survivors and nonsurvivors (Fig. 2b). Interestingly, we found that these metabolite biomarkers are enriched in several death-related metabolic pathways (DRMPs). In detail, in sepsis nonsurvivors, lysophospholipid metabolism and phospholipid metabolism were significantly downregulated in nonsurvivors compared to the survivors (Fig. 2b and Additional file 1: Table S7). Acylcarnitines were observed to be increased dramatically in the plasma and serum of the sepsis nonsurvivors, indicating the downregulation of fatty acid oxidation. In addition, metabolites in the TCA cycle were increased in sepsis nonsurvivors. Together, these findings indicated the mitochondrial dysfunction in sepsis nonsurvivors. Furthermore, branched-chain amino acid metabolism, amino-sugar metabolism, and eicosanoids were dramatically upregulated in sepsis nonsurvivors (Fig. 2b).

### Meta-analysis of AUROCs

ROC curve analysis is an effective method for evaluating the accuracy of diagnostic tests in modern medicine. Out of 16 studies enrolled, 12 conducted ROC curve analysis using the biomarker metabolites and six performed the direct comparisons between metabolite biomarkers and traditional scores (Additional file 1: Table S8). We next pooled the AUROCs from each study and performed a meta-analysis to analyze the prediction accuracy for sepsis prognosis by metabolomics. The sensitivity analysis revealed that the overall AUROC estimation and conclusions were not affected by each study.

#### Prediction performance of combined metabolite biomarkers without summarizing pathways

**Fig. 3:**
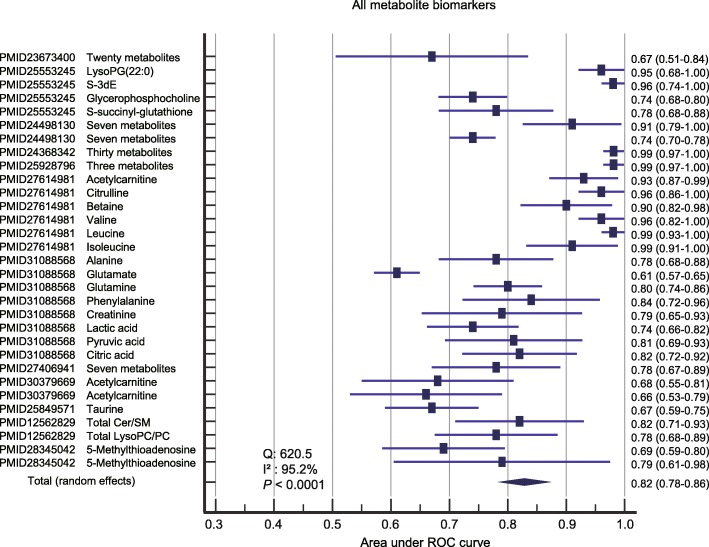
The pooled AUROC for the prediction accuracy of sepsis death using metabolite biomarkers without summarizing the pathways. LysoPG, lysophosphatidylglycerol; S-3dE, S-(3-methylbutanoyl)-dihydrolipoamide-E; Cer, ceramides; LysoPC, lysophosphatidylcholine; PC, phospholipids

We first performed the meta-analysis of combined biomarker metabolites without summarizing pathways. Visual inspection of the funnel plot of the included studies revealed significant asymmetry (Additional file 1: Figure S2). The random-effect model was selected for this meta-analysis due to the heterogeneity (*Q* = 620.5, *I*^2^ = 95.2%, and *P* < 0.0001). The combined metabolite biomarkers yielded a pooled AUROC of 0.82 (95% CI 0.78–0.86, *P* < 0.001) (Fig. 3).

#### Prediction performance of each DRMP

We then investigated the prediction accuracy of each DRMP on sepsis outcome, including lysophospholipid metabolism, amino acid, and mitochondrial metabolism.

**Fig. 4:**
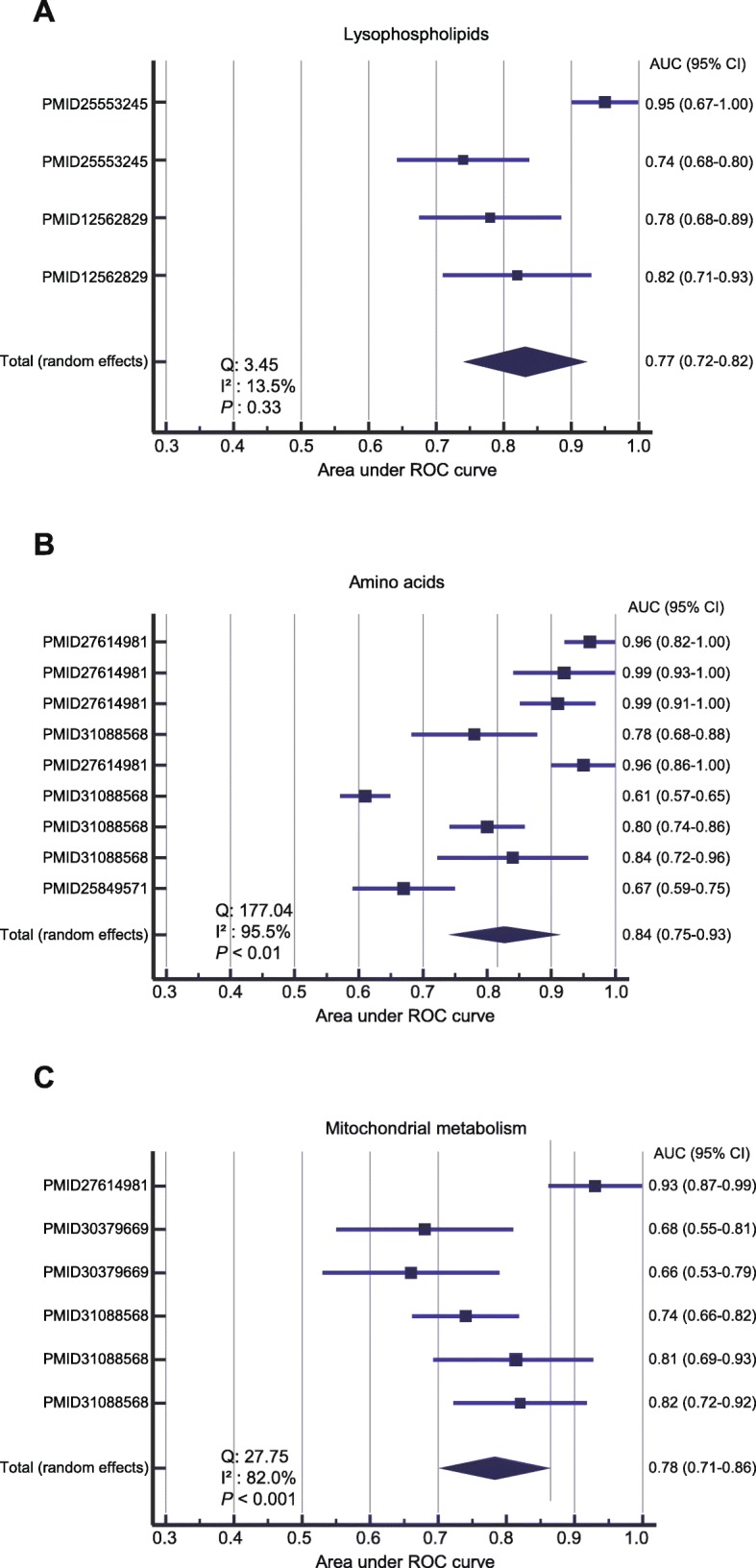
The AUROC for the prediction accuracy of sepsis death using metabolites from **a** lysophospholipid metabolism, **b** amino acid, and **c** mitochondrial metabolism. Results were presented as individual and pooled AUROC and 95% CI

*Lysophospholipid metabolism*. There was no significant publication bias for the studies reporting lysophospholipids based on the visualization of the funnel plot (Additional file 1: Figure S3). Cochran’s *Q* was 3.45, and Higgins’ *I*^2^ was 13.5% (*P* = 0.33). The fixed-effect model was then used for this analysis. Meta-analysis showed a pooled AUROC of 0.77 (95% CI 0.72–0.82) for lysophospholipid metabolism (Fig. 4a).

*Amino acid metabolism*. Visual inspection of the funnel plot revealed significant asymmetry, suggesting the presence of potential publication bias in the studies using amino acids as biomarkers for sepsis mortality prediction (Additional file 1: Figure S4). Strong evidence of heterogeneity between the included studies was observed (*Q* = 177.04, *I*^2^ = 95.5%, and *P* < 0.01). The random-effect model was then chosen for the meta-analysis. Metabolites from amino acid metabolism produced a combined AUROC of 0.84 (95% CI 0.75–0.93) (Fig. 4b).

*Mitochondrial metabolism*. The funnel plot analysis indicated that there was no significant publication bias for the studies using metabolite biomarkers from mitochondrial metabolism (Additional file 1: Figure S5). Cochran’s *Q* was 27.75, and Higgins’ *I*^2^ was 82.0% (*P* < 0.001). We then used the random-effect model for the analysis. Meta-analysis showed a pooled AUROC of 0.78 (95% CI 0.71–0.86) for the prediction of sepsis death (Fig. 4c).

#### Prediction performance of the combined DRMPs

**Fig. 5:**
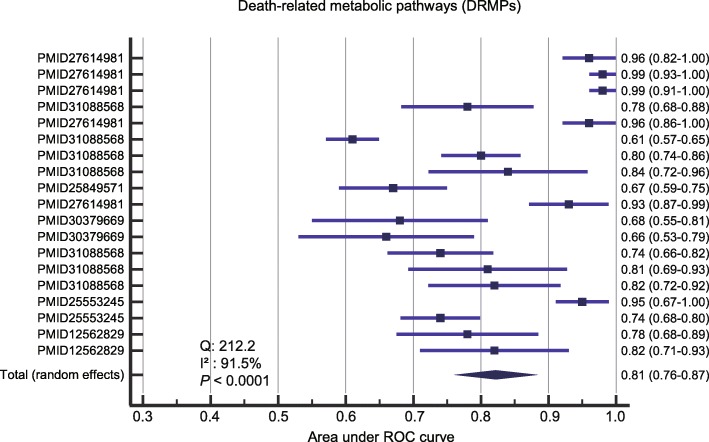
The pooled AUROCs for the prediction accuracy of sepsis death using DRMPs. The DRMPs were lysophospholipid, amino acid, and mitochondrial metabolism. Results were presented as individual and pooled AUROCs and 95% CI

We next investigated the performance of metabolite biomarkers from combined death-related metabolic pathways. Funnel plot analysis revealed no obvious publication bias (Additional file 1: Figure S6). We observed significant evidence of heterogeneity between the included studies (*Q* = 212.2, *I*^2^ = 91.5%, and *P* < 0.0001), and the random-effect model was then chosen for the meta-analysis. A merged AUROC of 0.81 (95% CI 0.76–0.87) was obtained using the combined DRMPs (Fig. 5).

**Fig. 6:**
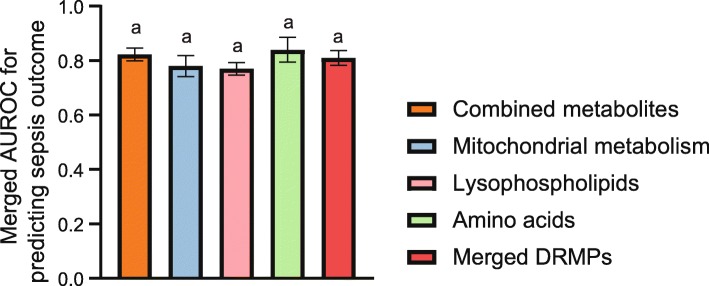
The comparison of the pooled AUROCs for the prediction accuracy of sepsis death using different biomarkers. One-way ANOVA was performed, and columns indexed by the same letter indicated that the differences are not significant (*P* > 0.05)

We compared the prediction accuracy for sepsis outcomes using different biomarkers. As shown in Fig. 6, the combined DRMPs have the similar prediction accuracy as merged metabolite biomarkers, and there was no significant difference in AUROCs between two approaches (*P* > 0.05).

### Validation of the meta-analysis using a prospective metabolomic study

**Fig. 7:**
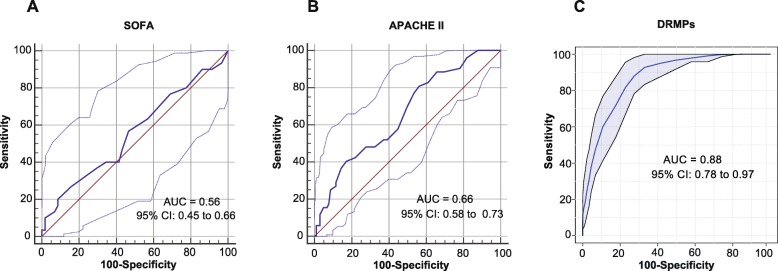
ROC analysis showed the prediction accuracy of sepsis death using **a** SOFA scores. **b** APACHE II scores. **c** DRMPs in the validation cohort. The multi-biomarkers used for ROC analysis were isoleucine (amino acid), alanine (amino acid), acetylcarnitine (mitochondrial metabolism), lactic acid (mitochondrial metabolism), pyruvic acid (mitochondrial metabolism), LysoPG (22:0) (lysophospholipids metabolism), and LysoPC (24:0). The ROC curve was generated by Monte Carlo cross-validation of random forest models. Repeated random cross-validation (rdCV) and permutation test were used for internal validation of the classification model

Finally, we conducted a prospective clinical study to confirm our findings in the meta-analysis. Metabolomic analysis was performed using the plasma samples collected from 188 sepsis patients, including 134 survivors and 54 nonsurvivors. The patients’ characteristics are listed in Table 2. The 3D PLS-DA plot revealed a distinct separation of the plasma metabolomic profiles between sepsis survivors and nonsurvivors (Additional file 1: Figure S7). We selected seven metabolites from the identified DRMPs including isoleucine (amino acid), alanine (amino acid), acetylcarnitine (mitochondrial metabolism), lactic acid (mitochondrial metabolism), pyruvic acid (mitochondrial metabolism), LysoPG (22:0) (lysophospholipid metabolism), and LysoPC (24:0) (lysophospholipid metabolism) as the potential biomarkers for sepsis outcome prediction. Interestingly, these metabolites were all statistically different between sepsis survivors and nonsurvivors, according to the Mann-Whitney *U* test (*P* < 0.05, 0.01, or 0.001, Additional file 1: Figure S8). ROC analysis of selected metabolites based on Monte Carlo cross-validation of random forest models yielded an AUROC of 0.88 (95% CI 0.78–0.97), which was significantly higher than the SOFA score (AUROC of 0.56, 95% CI 0.45–0.66) and APACHE II score (AUROC of 0.66, 95% CI 0.58–0.83) (Fig. 7). The accuracy, sensitivity, and specificity for the prediction of sepsis death were 80.1% (95% CI 69.2–88.0%), 80.4% (95% CI 66.9–89.4%), and 78.8% (95% CI 62.3–89.3%), respectively (Additional file 1: Table S9). The negative likelihood ratio (NLR), positive likelihood ratio (PLR), and diagnostic odds ratio (DOR) were 0.25 (95% CI 0.13–0.46), 3.79 (95% CI 1.93–7.43), and 15.2 (95% CI 5.04–46.2), respectively (Additional file 1: Table S9). Cross-validation revealed the rdCV accuracy of 77.3% (Additional file 1: Table S9) and permutation *P* value (500 times) of 0.0002 (Additional file 1: Figure S9).

**Table 2:** The characteristics and clinical variables of patients in the prospective validation cohort

Clinical variable	Validation sepsis		*P* value
	Sepsis survivors	Sepsis nonsurvivors	
No.	134	54	
Age (years)	61.8 ± 18.5	67 ± 14.4	0.07
Gender (male %)	64.20%	61.10%	0.69
SOFA (median and IQR)	7 (5–12)	8 (6–13)	0.22
APACHE II (median and IQR)	18 (13–24)	22 (18–30)	0.001
MAP (mmHg)	75.2 ± 14.9	65.8 ± 15.2	0.001
PCT (median and IQR)	2.13 (0.26–15.9)	2.53 (0.74–36.5)	0.086
Pathogen			
*S. aureus* [*N* (%)]	13 (9.7%)	4 (7.4%)	0.62
*K. pneumoniae* [*N* (%)]	23 (17.2%)	4 (7.4%)	0.084
*E. coli* [*N* (%)]	9 (6.7%)	4 (7.4%)	0.86
Source of infection			
Respiratory [*N* (%)]	46 (34.3%)	23 (42.6%)	0.29
Abdominal [*N* (%)]	18 (13.4%)	7 (13.0%)	0.94
Urinary tract [*N* (%)]	6 (4.5%)	3 (5.6%)	0.75
Blood [*N* (%)]	33 (24.6%)	8 (14.8%)	0.14
Others [*N* (%)]	31 (23.1%)	13 (24.1%)	0.88
Comorbidities			
Diabetes mellitus [*N* (%)]	32 (23.9%)	16 (29.6%)	0.42
Cardiovascular disease [*N* (%)]	43 (32.1%)	23 (42.6%)	0.17
Malignancy [*N* (%)]	16 (11.9%)	11 (20.4%)	0.13
COPD [*N* (%)]	5 (3.7%)	2 (3.7%)	1
Chronic kidney disease [*N* (%)]	8 (6.0%)	2 (3.7%)	0.53
Chronic liver disease [*N* (%)]	8 (6.0%)	1 (1.9%)	0.24
Nervous system disease [*N* (%)]	20 (14.9%)	14 (25.9%)	0.077
Immunosuppression [*N* (%)]	14 (10.4%)	8 (14.8%)	0.4

Data were shown as mean ± standard deviation (SD) or median with interquartile range (IQR) or number with percentages depending on the distribution. *P* values were calculated by Student’s *t* test or Mann-Whitney *U* test or proportional *Z* test

*Abbreviations*: *SOFA* The Sequential Organ Failure Assessment, *APACHE II* Acute Physiology and Chronic Health Evaluation II, *MAP* mean arterial pressure, *PCT* procalcitonin, *S. aureus Staphylococcus aureus*, *K. pneumoniae Klebsiella pneumoniae*, *E. coli Escherichia coli*, *COPD* chronic obstructive pulmonary disease

## Discussion

Sepsis is one of the major causes of death in US hospitals. The identification of biomarkers that distinguish patients at high risk for poor outcomes will likely help the development of new treatment strategies. This meta-analysis of 21 cohorts with 1287 septic patients revealed that despite the heterogeneity of patients, there are unique metabolic signatures in sepsis nonsurvivors. Our study also showed that the analysis of DRMPs minimizes the discrepancies between metabolomic methods and is more practical than metabolite biomarkers themselves for prognostic prediction in patients with sepsis. To our knowledge, this is the first meta-analysis of the clinical utility of metabolomics for sepsis mortality prediction.

In this study, we made slight modifications for NOS based on the widely accepted guidelines for a reliable metabolomic study [24, 25]. For example, the sample size is a critical factor for metabolomic studies, and a good clinical metabolomic study generally requires at least 20 subjects per group to receive enough statistical power [49]. We thus replaced question 4 in the section of “selection” in original NOS with sample size check in the modified NOS (Additional file 1: Supplemental methods and Table S1 and S2).

**Fig. 8:**
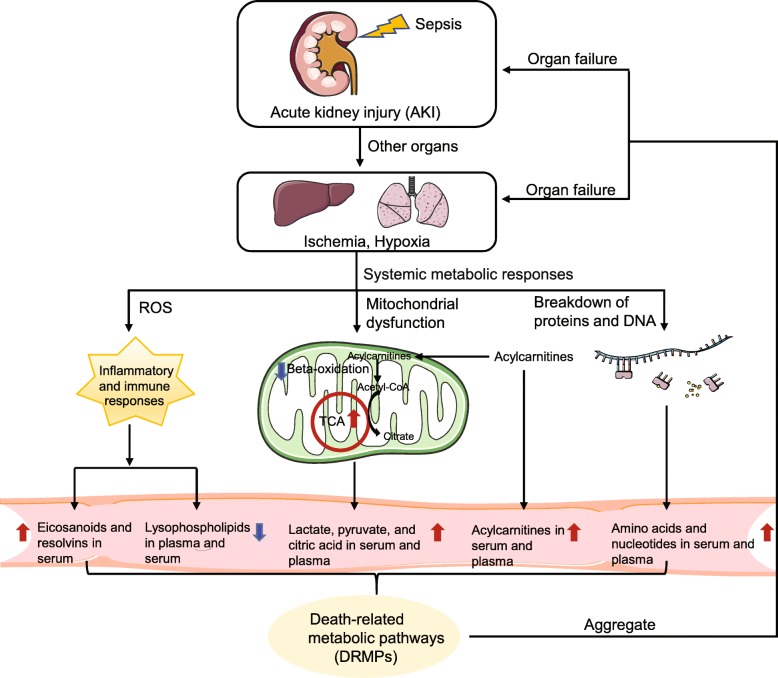
The death-related metabolic pathways (DRMPs) in the blood of sepsis nonsurvivors. Briefly, sepsis induces acute kidney injury (AKI), followed by ischemia and hypoxia in other organs such as the liver and lung. Acute respiratory distress syndrome (ARDS) in the lung contributes to the subsequent systemic metabolic responses, including inflammatory responses, defects of organ healing capability, mitochondrial dysfunction in energy production, and systemic uncontrolled proteolysis. These produce unique metabolic signatures in the blood of sepsis nonsurvivors, which can be measured by metabolomics. For example, the sharp increase of pro-inflammatory eicosanoids, the accumulation of TCA cycle metabolites (lactate, pyruvate, and citric acid), the increase of acylcarnitines and amino acids, and the significant reduction of lysophospholipids in the plasma and serum of sepsis nonsurvivors. The aggregate of these metabolites in DRMPs leads to multi-organ failure and death. This figure was created by ourselves

Our study identified death-related metabolic patterns in sepsis (Fig. 8). Briefly, infection-induced sepsis causes acute kidney injury (AKI) in the host [50], followed by ischemia and hypoxia in other organs such as the liver and lung. Acute respiratory distress syndrome (ARDS) in the lung contributes to the systemic metabolic responses [51, 52]. The aberrant metabolic responses of greatest importance to death in sepsis are mitochondrial dysfunction, breakdown of proteins and DNA, and uncontrolled inflammatory and immune responses, which produce DRMPs. The aggregation of metabolites in DRMPs leads to organ failure and eventually death.

The dramatic changes of metabolites in the serum and plasma of sepsis nonsurvivors include the increase of eicosanoids (eicosanoid metabolism), TCA cycle metabolites (lactate, pyruvate, and citric acid) (mitochondrial metabolism), acylcarnitines (mitochondrial metabolism), and amino acids (amino acid metabolism) and a sharp decrease of lysophospholipids (lysophospholipid metabolism).

### Eicosanoid “storms” and uncontrolled inflammation

Eicosanoids are essential lipid mediators involved in the onset of inflammation and the innate immune responses [53]. Our analysis revealed higher levels of circulating eicosanoids in sepsis nonsurvivors compared to the survivors. This observation was in line with the gene expression-based prognostic model developed by Sweeney and coauthors, in which genes from inflammation-related pathways were largely activated in sepsis nonsurvivors [54]. Both hyperinflammation and immune hypoactivation lead to patient morbidity and death [55, 56]. The combination of multi-omic results might provide a comprehensive view of death-related changes at the molecular level in sepsis nonsurvivors.

### Mitochondrial dysfunction in energy production

The accumulation of lactic acid, pyruvate, citric acid, and acylcarnitines in the serum and plasma of sepsis nonsurvivors indicates that death in sepsis might be associated with profound mitochondrial dysfunction in energy production. In contrast, sepsis survivors have better preservation of ATP, mitochondrial function, and biogenesis markers. Citric acid, lactic acid, and pyruvic acid are critical energetic substrates used by mitochondria for aerobic catabolism. Sepsis patients who ultimately die have poor aerobic catabolism due to mitochondrial dysfunction, displaying elevated concentrations of TCA cycle metabolites in plasma and serum. Acylcarnitines are essential for beta-oxidation of fatty acids and play essential roles in maintaining energy homeostasis in the human body. Acylcarnitines that are not utilized for energy production in fatty acid beta-oxidation due to the mitochondrial dysfunction will be reversely transported from mitochondria to the cytoplasm and then into the plasma and serum [57].

### Systemic uncontrolled proteolysis

In our analysis, we found that sepsis nonsurvivors usually have significantly higher levels of circulating branched-chain amino acids in their plasma and serum. This difference is likely caused by systemic uncontrolled proteolysis in sepsis. Recent studies had suggested uncontrolled proteolysis as the fundamental pathological mechanism in septic shock that contributes to cell injury and organ dysfunction [58, 59]. Additionally, the transcriptomic analysis showed the upregulation of genes associated with proteasome degradation in sepsis nonsurvivors [54].

### Profound defects of organ healing capability

In the nonsurvivors of sepsis patients, the levels of lysophospholipids were significantly lower than those in the survivors. Lysophosphatidic acid (LPA) had been reported to be overproduced in response to tissue injury, and it can promote healing in multiple organs such as the lung, skin, gastrointestinal tract, and cornea [60–62]. This suggested that the organ healing capability in nonsurvivors is lower than survivors.

### Prognostic model for mortality prediction based on DRMPs

Our analysis revealed limited overlap in the reported metabolite biomarkers for sepsis mortality prediction across studies. This is likely caused by the discrepancies between analytical methods used for metabolomic analysis in these studies, such as different analytical instruments, instrument sensitivity, and metabolome coverage, and different sample preparation approaches. Certain metabolite biomarkers might not be well measured in one metabolomic method compared to another. However, these biomarkers were enriched in several DRMPs, and the changes in DRMPs are highly consistent no matter what metabolites are measured in these pathways. The DRMPs and metabolite biomarkers had a similar accuracy for predicting sepsis outcomes. Therefore, the analysis of the combined changes in DRMPs might be more practical than specific metabolite biomarkers themselves for prognosis prediction in patients with sepsis.

### Limitations

Our study has some limitations. First, currently, there are no established guidelines for the experimental design, analytical procedures, and data analysis of clinical metabolomic studies. The data were obtained from studies that had different experimental designs, analytical platforms, and patients’ characteristics. The substantial heterogeneity among the studies may affect the interpretation of the results. Second, due to the lack of full datasets for metabolomic analysis of sepsis [63], we performed the meta-analysis using the reported metabolites. This approach is then susceptible to publication bias.

## Conclusions

Our meta-analysis of clinical metabolomic studies of sepsis prognosis reveals new roles of metabolism in sepsis-induced death and highlights the potential value of death-related metabolic pathways as the biomarkers in the prediction of sepsis mortality. These results will serve as a benchmark for future prognostic model development using metabolomics.

## Supplementary information

**Additional file 1: **Supplemental methods. Supplemental results. **Table S1.** The risk of bias assessment of included studies using NOS. **Table S2.** The risk of bias assessment of included studies using modified NOS. **Table S3.** The clinical metabolomic studies selected in the meta-analysis. **Table S4.** Dataset summary for the included studies. **Table S5.** Quality assessment of clinical metabolomic studies for the mortality prediction of sepsis. **Table S6.** The identified metabolites biomarkers for the prediction of sepsis death and their chemical classes, metabolic pathways, Log2 fold change and *P*-value. **Table S7.** The vote count, pooled *P*-value, and Log2 fold change of the significantly altered metabolic pathways. **Table S8.** Studies containing the direct comparisons of prediction accuracy for sepsis outcomes between metabolomics and traditional scores. **Table S9.** Prediction accuracy of selected 7-analyte from DRMPs for sepsis death. **Figure S1.** Venn diagram showing the poor overlap of reported metabolite biomarkers across the studies for sepsis mortality prediction. **Figure S2.** The visualization of publication bias by the funnel plot for studies using metabolite biomarkers. **Figure S3.** Assessment of publication bias by funnel plot for studies using lysophospholipids as biomarkers for sepsis outcome prediction. **Figure S4.** Evaluation of publication bias by funnel plot for studies using amino acids as biomarkers for sepsis outcome prediction. **Figure S5.** Assessment of publication bias by funnel plot for studies using metabolites from mitochondrial metabolism as biomarkers for sepsis outcome prediction. **Figure S6.** Assessment of publication bias by funnel plot for studies using metabolites from DRMPs as biomarkers for sepsis outcome prediction. **Figure S7.** 3-D PLS-DA revealed the distinct separation of the plasma metabolome of sepsis nonsurvivors from sepsis survivors. **Figure S8.** The significant differences of seven selected biomarkers from DRMPs between sepsis survivors and nonsurvivors. **Figure S9.** The permutation test for the validation of the classification model robustness.

## Data Availability

All data generated or analyzed during this study were included in this published article and its additional file.

## Abbreviations

AIDS: Acquired immune deficiency syndrome
AKI: Acute kidney injury
ANOVA: Analysis of variance
APACHE: Acute Physiology and Chronic Health Evaluation
ARDS: Acute respiratory distress syndrome
ATP: Adenosine triphosphate
AUROC: Area under receiver operating characteristic
CAS: Chemical Abstracts Service
Cer: Ceramides
CI: Confidence interval
CNKI: China National Knowledge Infrastructure
CQVIP: Chongqing VIP
DOR: Diagnostic odds ratio
DRMPs: Death-related metabolic pathways
EGDT: Early goal-directed therapy
FC: Fold change
HMDB: Human Metabolome Database
ICU: Intensive care unit
IRB: Institutional Review Board
KEGG: Kyoto Encyclopedia of Genes and Genomes
LPA: Lysophosphatidic acid
LysoPC: Lysophosphatidylcholine
LysoPG: Lysophosphatidylglycerol
MS: Mass spectrometry
NLR: Negative likelihood ratio
NOS: Newcastle-Ottawa Scale
PC: Phospholipids
PLR: Positive likelihood ratio
PLS-DA: Partial least squares discriminant analysis
QC: Quality control
RdCV: Repeated random cross-validation
ROC: Receiver operating characteristic
S-3dE: S-(3-methylbutanoyl)-dihydrolipoamide-E
SAPS: Simplified Acute Physiology Score
SM: Sphingomyelins
SOFA: Sequential Organ Failure Assessment
TCA: Tricarboxylic acid cycle
