# Supplementary material for: Prediction of sepsis mortality using metabolite biomarkers in the blood: a meta-analysis of death-related pathways and prospective validation
Source: BMC Med. 2020 Apr 15;18:83. doi: 10.1186/s12916-020-01546-5 (PMC7157979; doi:10.1186/s12916-020-01546-5)
Supplement: Supplementary file 1 — Additional file 1: Supplemental methods. Supplemental results. Table S1. The risk of bias assessment of included studies using NOS. Table S2. The risk of bias assessment of included studies using modified NOS. Table S3. The clinical metabolomic studies selected in the meta-analysis. Table S4. Dataset summary for the included studies. Table S5. Quality assessment of clinical metabolomic studies for the mortality prediction of sepsis. Table S6. The identified metabolites biomarkers for the prediction of sepsis death and their chemical classes, metabolic pathways, Log2 fold change and P-value. Table S7. The vote count, pooled P-value, and Log2 fold change of the significantly altered metabolic pathways. Table S8. Studies containing the direct comparisons of prediction accuracy for sepsis outcomes between metabolomics and traditional scores. Table S9. Prediction accuracy of selected 7-analyte from DRMPs for sepsis death. Figure S1. Venn diagram showing the poor overlap of reported metabolite biomarkers across the studies for sepsis mortality prediction. Figure S2. The visualization of publication bias by the funnel plot for studies using metabolite biomarkers. Figure S3. Assessment of publication bias by funnel plot for studies using lysophospholipids as biomarkers for sepsis outcome prediction. Figure S4. Evaluation of publication bias by funnel plot for studies using amino acids as biomarkers for sepsis outcome prediction. Figure S5. Assessment of publication bias by funnel plot for studies using metabolites from mitochondrial metabolism as biomarkers for sepsis outcome prediction. Figure S6. Assessment of publication bias by funnel plot for studies using metabolites from DRMPs as biomarkers for sepsis outcome prediction. Figure S7. 3-D PLS-DA revealed the distinct separation of the plasma metabolome of sepsis nonsurvivors from sepsis survivors. Figure S8. The significant differences of seven selected biomarkers from DRMPs between sepsis survivors and nonsurvivors. Fi [file 12916_2020_1546_MOESM1_ESM.docx]

**Additional file 1**

**Prediction of sepsis mortality using metabolite biomarkers in the blood: a meta-analysis of death-related pathways and prospective validation**

Jing Wang^1,2^, Yizhu Sun^1^, Shengnan Teng^1^, Kefeng Li^2*^

1. Department of Critical Care Medicine, Yantai Yuhuangding Hospital, Yantai, Shandong 264000, China
2. School of Medicine, University of California, San Diego, CA, 92103, United States

*Corresponding author: [kli@ucsd.edu](mailto:kli@ucsd.edu)

**Table of Contents**

[**Supplemental methods** 3](#_Toc29388174)

[**Supplemental results** 10](#_Toc29388175)

[**Table S1 The risk of bias assessment of included studies using NOS** 12](#_Toc29388176)

[**Table S2 The risk of bias assessment of included studies using modified NOS** 13](#_Toc29388177)

[**Table S3 The clinical metabolomic studies selected in the meta-analysis** 14](#_Toc29388178)

[**Table S4 Dataset summary for the included studies** 15](#_Toc29388179)

[**Table S5 Quality assessment of clinical metabolomic studies for the mortality prediction of sepsis** 17](#_Toc29388180)

[**Table S6 The identified metabolites biomarkers for the prediction of sepsis death and their chemical classes, metabolic pathways, Log2 fold change and *P*-value** 19](#_Toc29388181)

[**Table S7 The vote count, pooled *P*-value, and Log2 fold change of the significantly altered metabolic pathways** 36](#_Toc29388182)

[**Table S8 Studies containing the direct comparisons of prediction accuracy for sepsis outcomes between metabolomics and traditional scores** 38](#_Toc29388183)

[**Table S9 Prediction accuracy of selected 7-analyte from DRMPs for sepsis death** 39](#_Toc29388184)

[**Figure S1 Venn diagram showing the poor overlap of reported metabolite biomarkers across the studies for sepsis mortality prediction** 40](#_Toc29388186)

[**Figure S2 The visualization of publication bias by the funnel plot for studies using metabolite biomarkers** 41](#_Toc29388187)

[**Figure S3 Assessment of publication bias by funnel plot for studies using lysophospholipids as biomarkers for sepsis outcome prediction** 42](#_Toc29388188)

[**Figure S4 Evaluation of publication bias by funnel plot for studies using amino acids as biomarkers for sepsis outcome prediction** 43](#_Toc29388190)

[**Figure S5 Assessment of publication bias by funnel plot for studies using metabolites from mitochondrial metabolism as biomarkers for sepsis outcome prediction** 44](#_Toc29388191)

[**Figure S6 Assessment of publication bias by funnel plot for studies using metabolites from DRMPs as biomarkers for sepsis outcome prediction** 45](#_Toc29388192)

[**Figure S7 3-D PLS-DA revealed the distinct separation of the plasma metabolome of sepsis nonsurvivors from sepsis survivors** 46](#_Toc29388193)

[**Figure S8 The significant differences of 7 selected biomarkers from DRMPs between sepsis survivors and nonsurvivors** 47](#_Toc29388194)

[**Figure S9 The permutation test for the validation of the classification model robustness** 48](#_Toc29388196)

[**PRISMA checklist** 49](#_Toc29388198)

# **Supplemental methods**

**Search strategy used in the current systematic review and meta-analysis**

***Sepsis***

1. Severe Sepsis
2. Sepsis, Severe
3. Pyemia
4. Pyemias
5. Pyohemia
6. Pyohemias
7. Pyaemia
8. Pyaemias
9. Septicemia
10. Septicemias
11. Poisoning, Blood
12. Blood Poisoning
13. Poisonings, Blood
14. Bacteremia
15. Endotoxemia
16. Fungemia
17. Candidemia
18. Parasitemia
19. Viremia
20. Sepsis [MeSH]
21. OR/1 - 20

***Metabolomics***

1. Metabolomic
2. Metabonomics
3. Metabonomic
4. Metabolomics [MeSH]
5. OR/22 - 25

***Combined search:*** #21 AND #26

***Legend:*** MeSH indicates Medical Subject Heading in MEDLINE

***Records identified through database searching:***

PubMed: 246

EMBASE: 373

China National Knowledge Infrastructure: 70

Wanfang Data: 510

CQVIP: 11

The Cochrane Library: 8

Web of Science: 596

**Data extraction and quality assessment**

All candidate articles were independently evaluated, and the information was extracted by two authors (J.W. and S.T.). The full-texts were retrieved for review if the articles that could not be categorized based on title and abstract alone. If disagreement occurred, a third author (Y.S.) resolved discrepancies by discussion and adjudication. For each study, the following items were extracted: first author, year of publication, country, type of patients, total number of cases and gender, follow-up time, mortality, clinical scores (i.e. SOFA and APACHE II), the analytical platforms, metabolites with significant changes, metabolites fold change (FC, nonsurvivors/survivor), adjusted *P*-value, area under receiver operating characteristic (ROC) curve (AUROC) and validation.

The Newcastle-Ottawa Scale (NOS) has three parts: selection (0 - 4 points), comparability (0 - 2 points), and outcome assessment (0 - 3 points). We made slight modifications in the parts of selection and outcome assessment in original NOS based on the guidelines for a reliable metabolomic study.

| **NOS (Newcastle-Ottawa Scale) for Cohort study**  **Website:** http://www.ohri.ca/programs/clinical_epidemiology/oxford.asp | | |
| --- | --- | --- |
| Wells GA and his colleagues | **Selection**   1. Representativeness of the exposed cohort    1. truly representative of the average _______________ (describe) in the community    2. somewhat representative of the average ______________ in the community    3. selected group of users eg nurses, volunteers    4. no description of the derivation of the cohort 2. Selection of the non exposed cohort    1. drawn from the same community as the exposed cohort    2. drawn from a different source    3. no description of the derivation of the non exposed cohort 3. Ascertainment of exposure    1. secure record (eg surgical records)    2. structured interview    3. written self report    4. no description 4. Demonstration that outcome of interest was not present at start of study 5. yes 6. no   **Comparability**   1. Comparability of cohorts on the basis of the design or analysis    1. study controls for _____________ (select the most important factor)    2. study controls for any additional factor (This criteria could be modified to indicate specific control for a second important factor.)   **Outcome**   1. Assessment of outcome    1. independent blind assessment    2. record linkage    3. self report    4. no description 2. Was follow-up long enough for outcomes to occur 3. yes (select an adequate follow-up period for outcome of interest) 4. no 5. Adequacy of follow up of cohorts 6. complete follow up - all subjects accounted for 7. subjects lost to follow up unlikely to introduce bias - small number lost - > ____ % (select an adequate %) follow up, or description provided of those lost) 8. follow up rate < ____% (select an adequate %) and no description of those lost 9. no statement | ☆  ☆  /  /  ☆  /  /  ☆  ☆  /  /  ☆  /  ☆  ☆  ☆  ☆  /  /  ☆  /  ☆  ☆  /  / |

Modified Newcastle-Ottawa risk of bias scoring guide

（1） Selection (0 - 4 points)

1. Sample representativeness

- 1 point: Representative of the average adult in the community.
- 0 point: Selected group of patients, e.g. only pediatric cohorts.

1. Selection of the non-exposed cohort

- 1 point: Drawn from the same community as the nonsurvivors cohort.
- 0 point: Drawn from a different source.

1. Ascertainment of exposure

- 1 point: Follow the standard sepsis diagnosis criteria with the secure record.
- 0 point: No description.

1. Sample size

- 1 point: The sample size was greater than or equal to 20 participants for each group.
- 0 point: The sample size was less than 20 subjects.
  1. Comparability (0 - 2 points)

1. Study controls for age/sex

- 1 point: Yes
- 0 point: No

1. Study controls for any additional factor.

- 1 point: Yes
- 0 point: No
  1. Outcome (0 - 3 points)

1. Assessment of outcome?

- 1 point: Independent blind assessment or record linkage.
- 0 point: No description.

1. Was follow-up long enough for the outcome to occur?

- 1 point: follow-up ≥ 28 days
- 0 point: follow-up < 28 days

1. Quality of statistics reporting in metabolomics.

- 1 point: The study reported the validation of statistic models for large-scale metabolomic dataset.
- 0 point: The study did not validate the statistic models using independent cohorts, other analytical approaches or statistical methods.

Because of the inconsistency of the names for the same reported metabolites between publications, especially for the nomenclature of lipids, we then used the software OpenRefine and the ID conversion tool in MetaboAnalyst 4.0 to match the names in the publications to the names in HMDB or PubChem. We removed the ambiguity by adding the identifiers to each metabolite if available (CAS, HMDB or KEGG). We performed cross-data quality checks between reviewers at each step and reviewed all the included references after dataset construction.

**Validation of meta-analysis results using a prospective cohort metabolomic study**

This prospective cohort study was approved by the Institutional Review Board (IRB) of Yantai Yuhuangding Hospital ([2018]11) and registered on the Chinese Clinical Trial Registry (Registry ID: ChiCTR1800015321). All the protocols were conformed to the World Medical Association Declaration of Helsinki-Ethical Principles for Medical Research Involving Human Subjects. Briefly, the diagnostic evaluation was performed on admission and patients who met the Third International Consensus Definitions for Sepsis and Septic Shock were eligible for selection. The exclusion standards included the following factors: (1) younger than 18 years or older than 85 years of age; (2) diabetes and other metabolic-related diseases; (3) AIDS; (4) pregnant women; (5) incomplete clinical data. A total of 188 patients were enrolled between June 2017 and May 2018, and written permission was obtained from all the patients or their guardians. SOFA and APACHE II scores were calculated during the first 24 h of admission. Heparinized plasma samples were also collected at the time of ICU administration and stored at -80 ºC till extraction. Infection status and outcome through day 28 were adjudicated, and survival/death was the primary outcome. A atotal of 134 survivors and 54 nonsurvivors were included in our study.

Metabolite extraction and metabolomic analysis were performed as described before. Briefly, 90 µL of plasma was mixed with 10 µL of stable isotope internal standard mix. Macromolecules (protein, DNA, RNA, glycans, etc.) were precipitated by adding 400 µL of prechilled (-20°C), MEOH: ACN (50:50, v/v). The mixture was incubated on ice for 10 min and centrifuged at 16,000 g for 10 min at 4 ºC. The supernatant was transferred to new tubes and stored at -80 ºC for LC-MS/MS analysis.

Ten µL of the extract was injected and separated by a Shimadzu LC-20AD UHPLC on a 250 mm × 2.0 mm, 5 µm Luna NH_2_ HPLC column (Phenomenex, CA). The mobile phase was: mobile phase A was 95% H_2_O with 20 mM ammonium formate and 5% acetonitrile (pH 4). Mobile phase B was 100% acetonitrile. The gradient was as follows: 0 min-95% B, 3 min-95% B, 3.1 min-85% B, 6.0 min-85% B, 6.1 min-75% B, 10 min-75% B, 15 min-0% B, 25 min-0%, 26 min-95% B, 31 min-end. The flow rate was 200 µL/min.

MS/MS analysis was performed by scheduled multiple reaction monitoring (sMRM) in both negative and positive mode with rapid polarity switching. A total of 620 metabolites were targeted. The values for MRM pairs, declustering potential (DP), entrance potential (EP), collision energy (CE), and collision cell exit potential (CXP) were determined and optimized for each MRM for each metabolite.

The chromatographic peaks were identified by using MultiQuant (v3.0, AB SCIEX), confirmed by manual inspection, and the peak areas were integrated. Data were log2 transformed before statistical analysis. Partial least squares discriminant analysis (PLS-DA) was conducted in MetaboAnalyst 4.0. Seven metabolites including isoleucine (amino acids), alanine (amino acids), acetylcarnitine (mitochondrial metabolism), lactic acid (mitochondrial metabolism), pyruvic acid (mitochondrial metabolism), lysoPG (22:0) (lysophospholipids metabolism) and lysoPC (24:0) (lysophospholipids metabolism) were selected based on the results of meta-analysis. ROC curve analysis with a random forest model was performed using MetaboAnalyst 4.0 to evaluate the performance of selected metabolites for the prediction of sepsis outcome. Repeated random cross-validation and permutation test were used for internal validation of the established classification model.

# **Supplemental results**

**Compilation and creation of the metabolomic dataset for the prognosis of sepsis**

Since the deposition of metabolomic data in publicly available repositories is currently not mandatory by scientific journals, the original data were not available for any of the metabolomic studies related to the prognosis of sepsis in our analysis. To confirm this, we also searched the common publicly repositories for metabolomic data such as Metabolights and Metabolomics Workbench, and no relevant metabolomic dataset was found too. In contrast, the full datasets for other omic analyses, such as genomics, transcriptomics, and metagenomics analysis, are usually accessible through repositories. To overcome this circumstance, we systematically reviewed the literature to identify all clinical metabolomic studies containing the prognosis of sepsis published before July 2019 and created the comprehensive datasets for meta-analysis by retrospective manual curation.

For all studies, we extracted data on key methodological parameters such as the number of patients, gender, age, the outcome of prognosis, SOFA score, biological fluids collected, and the analytical platforms. We also extracted the critical information for all reported metabolites such as fold changes and adjusted *P*-value. During curation, we found that the metabolomic data reporting is largely inconsistent in terms of metabolites names, especially for the nomenclature of lipids. We then used the software OpenRefine and the ID conversion tool in MetaboAnalyst 4.0 to match the names in the publications to the names in HMDB or PubChem. We removed the ambiguity by adding the identifiers to each metabolite if available (CAS, HMDB, or KEGG). We performed data quality checks at each step of the dataset construction and reviewed all the included references post-dataset construction.

**Quality assessment of clinical metabolomic studies for sepsis mortality prediction**

*Quality assurance* (QA) *and bias evaluation*

QA addresses the experimental design and systematic activities implemented before samples were collected to provide confidence that quality requirements will be fulfilled. QA is the prerequisite for the success of a clinical metabolomic study. Here, we first evaluated the risk of the bias caused by experimental design for the selected clinical studies. The modified Newcastle-Ottawa quality assessment criteria for cohort studies were used (Supplemental Methods). The form assesses sample representativeness and size, comparability between survivors and nonsurvivors (age, sex, and other factors controlled), ascertainment of outcomes, and the quality of statistics reporting in metabolomics.

*Analytical platforms and metabolomic approaches*

Both untargeted and targeted metabolomic approaches had been applied to analyze the metabolic signatures of sepsis in plasma or serum that could be used for the prediction of death in sepsis (Table S5). The analytical platforms used for the analysis included LC-MS/MS (Triple quadrupole), LC-LTQ-orbitrap-MS, LC-Q-orbitrap, DSQ GC-MS and NMR.

*Quality control for metabolomic analysis*

Quality control is essential for obtaining reliable metabolomic data. Metabolomic analysis of hundreds of metabolites might be a challenging task for quality control. The metabolite coverage, retention time, and abundance have to be carefully monitored using the quality controls. Out of 16 studies included in our meta-analysis, 12 (75%) had quality controls (Table S5). For targeted metabolomic analysis, the use of internal standards has become the common practice for ensuring data quality, and the common guidelines are adapted from the guidance published by the FDA. For untargeted analysis, the use of pooled QC samples is a practical approach for quality control. The pooled QC samples were used in all MS-driven untargeted metabolic profiling studies of the prognosis of sepsis. However, QC samples and quality control protocols were not described in any NMR-based clinical metabolomic analysis of the prognosis of sepsis.

*Clinical or orthogonal validation*

The metabolomic analysis produces large datasets with high complexity and heterogeneity, and statistical analysis of such data is prone to errors (“false positives” or “false negative”). All *P*-values should be adjusted under multiple testing conditions to prevent over-optimistic interpretation. In addition, the results have to be better validated using independent cohorts, or verified using other models or by using other omic technologies such as proteomics and transcriptomics. However, only 6 out of 16 studies in our analysis reported one or multiple validations, including 2 studies with mice models, 4 with independent cohorts and 1 with proteomics (Table S5). Therefore, the meta-analysis here is useful to further validate the findings of individual studies.

# **Table S1 The risk of bias assessment of included studies using NOS**

| **Pubmed ID** | **NOS** | 1 | 2 | 3 | 4 | 5 | 6 | 7 | 8 | 9 |
| --- | --- | --- | --- | --- | --- | --- | --- | --- | --- | --- |
| 12562829 | 7 | √ | √ | √ | √ |  |  | √ | √ | √ |
| 23673400 | 8 |  | √ | √ | √ | √ | √ | √ | √ | √ |
| 23884467 | 9 | √ | √ | √ | √ | √ | √ | √ | √ | √ |
| 24368342 | 7 | √ | √ | √ | √ | √ | √ | √ |  |  |
| 24498130 | 9 | √ | √ | √ | √ | √ | √ | √ | √ | √ |
| 25553245 | 8 | √ | √ | √ | √ | √ | √ | √ |  | √ |
| 25849571 | 9 | √ | √ | √ | √ | √ | √ | √ | √ | √ |
| 25887472 | 9 | √ | √ | √ | √ | √ | √ | √ | √ | √ |
| 25928796 | 7 | √ | √ | √ | √ | √ | √ | √ |  |  |
| 26847922 | 9 | √ | √ | √ | √ | √ | √ | √ | √ | √ |
| 27406941 | 7 | √ | √ | √ | √ |  |  | √ | √ | √ |
| 27614981 | 8 | √ | √ | √ | √ | √ | √ | √ |  | √ |
| 27632672 | 8 | √ | √ | √ | √ | √ | √ | √ |  | √ |
| 28345042 | 9 | √ | √ | √ | √ | √ | √ | √ | √ | √ |
| 30379669 | 9 | √ | √ | √ | √ | √ | √ | √ | √ | √ |
| 31088568 | 8 | √ | √ | √ | √ | √ | √ | √ |  | √ |

# **Table S2 The risk of bias assessment of included studies using modified NOS**

| **Pubmed ID** | **Modified**  **NOS** | 1 | 2 | 3 | 4 | 5 | 6 | 7 | 8 | 9 |
| --- | --- | --- | --- | --- | --- | --- | --- | --- | --- | --- |
| 12562829 | 6 | √ | √ | √ | √ |  |  | √ | √ |  |
| 23673400 | 7 |  | √ | √ |  | √ | √ | √ | √ | √ |
| 23884467 | 9 | √ | √ | √ | √ | √ | √ | √ | √ | √ |
| 24368342 | 6 | √ | √ | √ |  | √ | √ | √ |  |  |
| 24498130 | 9 | √ | √ | √ | √ | √ | √ | √ | √ | √ |
| 25553245 | 6 | √ | √ | √ |  | √ | √ | √ |  |  |
| 25849571 | 7 | √ | √ | √ |  | √ | √ | √ | √ |  |
| 25887472 | 8 | √ | √ | √ | √ | √ | √ | √ | √ |  |
| 25928796 | 6 | √ | √ | √ |  | √ | √ | √ |  |  |
| 26847922 | 7 | √ | √ | √ |  | √ | √ | √ | √ |  |
| 27406941 | 6 | √ | √ | √ | √ |  |  | √ | √ |  |
| 27614981 | 7 | √ | √ | √ | √ | √ | √ | √ |  |  |
| 27632672 | 6 | √ | √ | √ |  | √ | √ | √ |  |  |
| 28345042 | 8 | √ | √ | √ |  | √ | √ | √ | √ | √ |
| 30379669 | 9 | √ | √ | √ | √ | √ | √ | √ | √ | √ |
| 31088568 | 8 | √ | √ | √ | √ | √ | √ | √ |  | √ |

# **Table S3 The clinical metabolomic studies selected in the meta-analysis**

| **PubMed ID** | **Journal name** | **First Author** | **Year** | **Study region** |
| --- | --- | --- | --- | --- |
| PMID12562829 | J Lipid Res | Wolfgang Drobnik | 2003 | Germany |
| PMID23673400 | Intensive Care Med | Christopher W. Seymour | 2013 | USA |
| PMID23884467 | Sci Transl Med | Raymond J. Langley | 2013 | USA |
| PMID24368342 | Crit Care Med | Beata Mickiewicz | 2014 | Canada |
| PMID24498130 | PLoS One | Angela J. Rogers | 2014 | USA |
| PMID25553245 | BMJ Open Respir Res | Longxiang Su | 2014 | China |
| PMID25849571 | PLoS One | Longxiang Su | 2015 | China |
| PMID25887472 | Crit Care | Kubra Kamisoglu | 2015 | USA |
| PMID25928796 | Crit Care | Beata Mickiewicz | 2015 | Canada |
| PMID26847922 | Sci Rep | Manuela Ferrario | 2016 | Italy |
| PMID27406941 | JPEN J Parenter Enteral Nutr | Kris M. Mogensen | 2017 | USA |
| PMID27614981 | Anal Bioanal Chem | Zhicheng Liu | 2016 | France |
| PMID27632672 | Crit Care Med | Jesmond Dalli | 2017 | USA |
| PMID28345042 | Sci Adv | Liuyang Wang | 2017 | USA |
| PMID30379669 | Crit Care Med | Kuei-Pin Chung | 2019 | China |
| PMID31088568 | Crit Care | Zhicheng Liu | 2019 | France |

Note: The table was sorted by PubMed ID.

# **Table S4 Dataset summary for the included studies**

| **Categories** | **Count No.** |
| --- | --- |
| Studies | 16 |
| Cohorts | 21 |
| Metabolite measurements (comparisons) | 2509 |
| Sepsis survivors | 851 |
| Sepsis nonsurvivors | 436 |
| Total patients | 1287 |

Cohorts: The number of cohorts included in the studies.

Metabolites measures (comparisons): The total number of “sepsis nonsurvivors vs sepsis survivors”

metabolites measures.

Total patients: The number of patients (sepsis survivors and nonsurvivors) included in the studies.

# **Table S5 Quality assessment of clinical metabolomic studies for the mortality prediction of sepsis**

| **PubMed ID** | **NOS** | **Modified** **NOS** | **Analytical platform** | **Metabolomic approach** | **Metabolites identified** | **Analytical quality control** | **Validation** | **Rural data deposit** |
| --- | --- | --- | --- | --- | --- | --- | --- | --- |
| PMID12562829 | 7 | 6 | LC-MS/MS | Targeted | 30 | Internal standards | N/A | N/A |
| PMID23673400 | 8 | 7 | LC-LTQ-orbitrap-MS and DSQ GC-MS | Untargeted | 423 | Pooled QC samples | Validation using the mice model | N/A |
| PMID23884467 | 9 | 9 | LC-Q-orbitrap-MS, DSQ GC-MS and LC-MS/MS | Untargeted and targeted | 439 | Pooled QC samples | Two validation cohorts and extra validation by proteomics | N/A |
| PMID24368342 | 7 | 6 | NMR | Untargeted | 60 | N/A | N/A | N/A |
| PMID24498130 | 9 | 9 | LC-Q-orbitrap-MS and DSQ GC-MS | Untargeted | 439 | Pooled QC samples | Validation cohort | N/A |
| PMID25553245 | 8 | 6 | LC-Q-orbitrap MS | Untargeted | 20 | N/A | N/A | N/A |
| PMID25849571 | 9 | 7 | LC-MS/MS | Targeted | 46 | Internal standards | N/A | N/A |
| PMID25887472 | 9 | 8 | LC-Q-orbitrap-MS and DSQ GC-MS | Untargeted | 366 | Pooled QC samples | N/A | N/A |
| PMID25928796 | 7 | 6 | NMR | Untargeted | 60 | N/A | N/A | N/A |
| PMID26847922 | 9 | 7 | LC-MS/MS | Targeted | 187 | Internal standards | N/A | N/A |
| PMID27406941 | 7 | 6 | LC-Q-orbitrap-MS and DSQ GC-MS | Untargeted | 281 | Pooled QC samples | N/A | N/A |
| PMID27614981 | 8 | 7 | LC-Q-orbitrap-MS | Untargeted | 42 | Pooled QC samples | N/A | N/A |
| PMID27632672 | 8 | 6 | LC-MS/MS | Targeted | 30 | Pooled QC samples | N/A | N/A |
| PMID28345042 | 9 | 8 | LC-MS/MS | Targeted | 1 | Pooled QC samples | Validation using the mice model | N/A |
| PMID30379669 | 9 | 9 | LC-MS/MS | Targeted | 41 | Internal standards | Multi-center validation cohort | N/A |
| PMID31088568 | 8 | 8 | NMR | Untargeted | 44 | N/A | Validation cohort | N/A |

NOS: Newcastle-Ottawa Scale; LC-MS/MS: Liquid chromatography with tandem mass spectrometry; LC-LTQ-orbitrap-MS: Liquid chromatography with linear trap quadrupole (LTQ) orbitrap mass spectrometry; DSQ GC-MS: Dual-Stage Quadrupole Gas chromatography-mass spectrometer; LC-Q-orbitrap-MS: Liquid chromatography with quadrupole orbitrap mass spectrometry; NMR: Nuclear magnetic resonance spectroscopy; QC: Quality control; N/A: Not available.

# **Table S6 The identified metabolites biomarkers for the prediction of sepsis death and their chemical classes, metabolic pathways, Log2 fold change and *P*-value**

| **PubMed ID** | **Matrix** | **Chemical classes** | **Metabolic pathways** | **HMDB ID** | **KEGG ID** | **Metabolites** | **Average Log2 of fold change (Nonsurvivors/**  **survivors)** | ***P*-value** |
| --- | --- | --- | --- | --- | --- | --- | --- | --- |
| PMID23673400 | Plasma | Cofactors and vitamins | Vitamin B6 metabolism | HMDB  0000017 | C00847 | 4-Pyridoxic acid | 2.652 | 0.001 |
| PMID23673400 | Plasma | Lipids | Steroid metabolism | HMDB  0000063 | C00735 | Cortisol | 1.632 | 0.004 |
| PMID23673400 | Plasma | Lipids | Bile acid metabolism | HMDB  0002581 | N/A | Taurocholic acid 3-sulfate | 1.519 | 0.001 |
| PMID23673400 | Plasma | Cofactors and vitamins | Nicotinate and nicotinamide metabolism | HMDB  0004193 | C05842 | N1-methyl-2-pyridone-5-carboxamide | 1.480 | 0.008 |
| PMID23673400 | Plasma | Carbohydrates | Amino-Sugar, Galactose, & Non-Glucose Metabolism | HMDB  0000765 | C00392 | Mannitol | 1.436 | 0.007 |
| PMID23673400 | Plasma | Organic acids and derivatives | Amino-Sugar, Galactose, & Non-Glucose Metabolism | HMDB  0060256 | C05411 | ​L-Xylonic acid | 1.415 | 0.004 |
| PMID23673400 | Plasma | Cofactors and vitamins | Vitamin E metabolism | HMDB  0060256 | N/A | Alpha-CEHC glucuronide | 1.188 | 0.002 |
| PMID23673400 | Plasma | Amino acids and derivatives | Urea cycle | HMDB  0000294 | C00086 | Urea | 1.101 | 0.008 |
| PMID23673400 | Plasma | Lipids | Bile acid metabolism | N/A | N/A | Glycocholenate sulfate | 1.054 | 0.001 |
| PMID23673400 | Plasma | Amino acids and derivatives | Tryptophan, Kynurenine, Serotonin, Melatonin Metabolism | HMDB  0000715 | C01717 | Kynurenic acid | 1.022 | 0.007 |
| PMID23673400 | Plasma | Organic acids and derivatives | Amino-Sugar, Galactose, & Non-Glucose Metabolism | HMDB  0000613 | N/A | Erythronic acid | 0.953 | 0.008 |
| PMID23673400 | Plasma | Amino acids and derivatives | Tryptophan, Kynurenine, Serotonin, Melatonin Metabolism | N/A | N/A | C-glycosyltryptophan | 0.908 | 0.007 |
| PMID23673400 | Plasma | Amino acids and derivatives | Microbiome Metabolism | N/A | N/A | N-acetyl serine | 0.639 | 0.007 |
| PMID23673400 | Plasma | Nucleotides and derivatives | Pyrimidine metabolism | HMDB  0000767 | C02067 | Pseudouridine | 0.601 | 0.003 |
| PMID23673400 | Plasma | Carbohydrates | Glycolysis and Gluconeogenesis Metabolism | HMDB  0000139 | C00258 | Glyceric acid | 0.543 | 0.007 |
| PMID23673400 | Plasma | Lipids | Fatty Acid Oxidation and Synthesis | HMDB  0060038 | N/A | 10Z-Heptadecenoic acid | 0.535 | 0.008 |
| PMID23673400 | Plasma | Organic acids and derivatives | Krebs cycle | HMDB  0000134 | C00122 | Fumaric acid | 0.485 | 0.005 |
| PMID23673400 | Plasma | Nucleotides and derivatives | Purine metabolism | HMDB  0003331 | C02494 | 1-Methyladenosine | 0.310 | 0.002 |
| PMID23673400 | Plasma | Lipids | Lysophospholipids metabolism | HMDB  0011477 | N/A | LysoPE(18:2) | -1.261 | 0.007 |
| PMID23673400 | Plasma | Lipids | Lysophospholipids metabolism | HMDB  0011475 | N/A | LysoPE(18:1) | -1.481 | 0.005 |
| PMID23884467 | Plasma | Lipids | Fatty Acid Oxidation and Synthesis | HMDB  0000378 | N/A | 2-Methylbutyroylcarnitine | 1.070 | 0.01 |
| PMID23884467 | Plasma | Lipids | Fatty Acid Oxidation and Synthesis | N/A | N/A | Decenoylcarnitine | 1.038 | 0.01 |
| PMID23884467 | Plasma | Lipids | Fatty Acid Oxidation and Synthesis | HMDB  0002013 | C02862 | Butyrylcarnitine | 0.684 | 0.01 |
| PMID23884467 | Plasma | Lipids | Fatty Acid Oxidation and Synthesis | HMDB  0000705 | N/A | Hexanoylcarnitine | 0.987 | 0.01 |
| PMID23884467 | Plasma | Organic acids and derivatives | Glycolysis and Gluconeogenesis Metabolism | HMDB  0000190 | C00186 | Lactic acid | 0.487 | 0.01 |
| PMID25553245 | Serum | Lipids | Lysophospholipids metabolism | N/A | N/A | LysoPG(22:0) | 0.753 | 0.005 |
| PMID25553245 | Serum | Amino acids and derivatives | Branch Chain Amino Acid Metabolism | HMDB  0006867 | C15975 | S-(3-methylbutanoyl)-dihydrolipoamide-E | 1.402 | 0.044 |
| PMID25553245 | Serum | Lipids | Phospholipids metabolism | HMDB  0000086 | C00670 | Glycerophosphocholine | 0.21 | 0.037 |
| PMID25553245 | Serum | Amino acids and derivatives | SAM, SAH, Methionine, Cysteine, Glutathione Metabolism | N/A | N/A | S-succinyl-glutathione | 0.638 | 0.013 |
| PMID24498130 | Plasma | Peptides | Gamma-Glutamyl and other Dipeptides | HMDB  0000594 | N/A | gamma-glutamylphenylalanine | 1.59 | 0.0017 |
| PMID24498130 | Plasma | Peptides | Gamma-Glutamyl and other Dipeptides | HMDB  0011741 | N/A | gamma-glutamyltyrosine | 1.28 | 0.001 |
| PMID24498130 | Plasma | Lipids | Lysophospholipids metabolism | HMDB  0010395 | N/A | LysoPC(20:4） | -1.51 | 0.0001 |
| PMID24498130 | Plasma | Lipids | Bile acid metabolism | HMDB  0000951 | C05465 | Taurochenodeoxycholic acid | 0.59 | 0.0014 |
| PMID24498130 | Plasma | Amino acids and derivatives | Tyrosine metabolism | N/A | N/A | 4-hydroxyphenyllactate | 1.09 | 0.0003 |
| PMID  24498130 | Plasma | Carbohydrates | Amino-Sugar, Galactose, & Non-Glucose Metabolism | HMDB  0000258 | C00089 | Sucrose | 0.35 | 0.0014 |
| PMID24498130 | Plasma | Amino acids and derivatives | Tryptophan, Kynurenine, Serotonin, Melatonin Metabolism | HMDB  0000684 | C00328 | Kynurenine | 0.46 | 0.0137 |
| PMID24368342 | Serum | Organic acids and derivatives | Microbiome metabolism | HMDB  0001873 | C02632 | Isobutyric acid | 0.740 | 0.0007 |
| PMID24368342 | Serum | Amino acids and derivatives | Tyrosine and Phenylalanine Metabolism | HMDB  0000159 | C00079 | Phenylalanine | 0.799 | 0.00012 |
| PMID24368342 | Serum | Organic acids and derivatives | Branch Chain Amino Acid Metabolism | HMDB  0000407 | N/A | 2-Hydroxy-3-methylbutyric acid | 1.151 | 0.0006 |
| PMID24368342 | Serum | Carbohydrates | Amino-Sugar, Galactose, & Non-Glucose Metabolism | HMDB  0000211 | C00137 | myo-Inositol | 1.770 | 0.00002 |
| PMID24368342 | Serum | Amino acids and derivatives | SAM, SAH, Methionine, Cysteine, Glutathione Metabolism | HMDB  0000562 | C00791 | Creatinine | 0.766 | 0.0004 |
| PMID24368342 | Serum | Amino acids and derivatives | Urea cycle | HMDB  0000294 | C00086 | Urea | 1.036 | 0.0005 |
| PMID24368342 | Serum | Lipids | Fatty Acid Oxidation and Synthesis | HMDB  0000201 | C02571 | Acetylcarnitine | 0.782 | 0.003 |
| PMID24368342 | Serum | Amino acids and derivatives | SAM, SAH, Methionine, Cysteine, Glutathione Metabolism | HMDB  0000064 | C00300 | Creatine | 1.556 | 0.003 |
| PMID24368342 | Serum | organic acids and derivatives | Glycolysis and Gluconeogenesis Metabolism | HMDB  0000190 | C00186 | Lactic acid | 0.632 | 0.004 |
| PMID24368342 | Serum | Organic acids and derivatives | Fatty Acid Oxidation and Synthesis | HMDB  0000357 | C01089 | 3-hydroxybutyric acid | 1.021 | 0.02 |
| PMID24368342 | Serum | Amino acids and derivatives | GABA, Glutamate, Arginine, Ornithine, Proline Metabolism | HMDB  0000162 | C00148 | Proline | 1 | 0.02 |
| PMID24368342 | Serum | Lipids | Fatty Acid Oxidation and Synthesis | HMDB  0000925 | C01104 | Trimethylamine N-oxide | 1.281 | 0.03 |
| PMID24368342 | Serum | Organic acids and derivatives | Krebs Cycle | HMDB  0000254 | C00042 | Succinic acid | 0.345 | 0.05 |
| PMID24368342 | Serum | Carbohydrates | Amino-Sugar, Galactose, & Non-Glucose Metabolism | HMDB  0000258 | C00089 | Sucrose | 0.895 | 0.05 |
| PMID24368342 | Serum | Amino acids and derivatives | Branch Chain Amino Acid Metabolism | HMDB  0000172 | C00407 | Isoleucine | -0.358 | 0.05 |
| PMID24368342 | Serum | Amino acids and derivatives | Bioamines and Neurotransmitter Metabolism | HMDB  0000641 | C00064 | Glutamine | -0.201 | 0.05 |
| PMID24368342 | Serum | Amino acids and derivatives | Branch Chain Amino Acid Metabolism | HMDB  0000161 | C00041 | Alanine | -0.322 | 0.05 |
| PMID24368342 | Serum | Amino acids and derivatives | Branch Chain Amino Acid Metabolism | HMDB  0000687 | C00123 | Leucine | -0.304 | 0.05 |
| PMID24368342 | Serum | Carbohydrates | Amino-Sugar, Galactose, & Non-Glucose Metabolism | HMDB  0000169 | C00159 | Mannose | -0.474 | 0.02 |
| PMID24368342 | Serum | Amino acids and derivatives | Branch Chain Amino Acid Metabolism | HMDB  0000182 | C00047 | Lysine | -0.269 | 0.02 |
| PMID24368342 | Serum | Amino acids and derivatives | Branch Chain Amino Acid Metabolism | HMDB  0000452 | C02356 | L-alpha-Aminobutyric acid | -0.474 | 0.02 |
| PMID24368342 | Serum | Amino acids and derivatives | SAM, SAH, Methionine, Cysteine, Glutathione Metabolism | HMDB  0001511 | C02305 | Phosphocreatine | -0.556 | 0.006 |
| PMID24368342 | Serum | Amino acids and derivatives | 1-Carbon, Folate, Formate, Glycine, Serine Metabolism | HMDB  0000123 | C00037 | Glycine | -0.377 | 0.005 |
| PMID24368342 | Serum | Amino acids and derivatives | SAM, SAH, Methionine, Cysteine, Glutathione Metabolism | HMDB  0000005 | C00109 | 2-Ketobutyric acid | -0.971 | 0.006 |
| PMID24368342 | Serum | Carbohydrates | Glycolysis and Gluconeogenesis Metabolism | HMDB  0000122 | C00031 | Glucose | -0.556 | 0.0004 |
| PMID24368342 | Serum | Amino acids and derivatives | 1-Carbon, Folate, Formate, Glycine, Serine Metabolism | HMDB  0000187 | C00065 | Serine | -0.621 | 0.006 |
| PMID24368342 | Serum | Amino acids and derivatives | Branch Chain Amino Acid Metabolism | HMDB  0000167 | C00188 | Threonine | -0.599 | 0.002 |
| PMID24368342 | Serum | Amino acids and derivatives | Bioamines and Neurotransmitter Metabolism | HMDB  0000148 | C00025 | Glutamic acid | -0.690 | 7.1×10-5 |
| PMID24368342 | Serum | Amino acids and derivatives | GABA, Glutamate, Arginine, Ornithine, Proline Metabolism | HMDB  0000517 | C00062 | Arginine | -0.690 | 0.0002 |
| PMID24368342 | Serum | Amino acids and derivatives | Branch Chain Amino Acid Metabolism | HMDB  0000883 | C00183 | Valine | -0.713 | 7.0×10-8 |
| PMID25928796 | Serum | Organic acids and derivatives | Branch Chain Amino Acid Metabolism | HMDB  0000407 | N/A | 2-Hydroxy-3-methylbutyric acid | 1.151 | 6.6×10-6 |
| PMID25928796 | Serum | Carbohydrates | Glycolysis and Gluconeogenesis Metabolism | HMDB  0000660 | C02336 | Fructose | 0.895 | 0.05 |
| PMID25928796 | Serum | Amines | Microbiome metabolism | HMDB  0000087 | C00543 | Dimethylamine | -0.786 | 0.0009 |
| PMID25887472 | Plasma | Amino acids and derivatives | Glycine, serine and threonine metabolism | HMDB  0000532 | N/A | N-acetylglycine | 1.070 | 0.002 |
| PMID25887472 | Plasma | Carbohydrates | Amino-Sugar, Galactose, & Non-Glucose Metabolism | HMDB  0000098 | C00181 | Xylose | 1.202 | 0.001 |
| PMID25887472 | Plasma | Amino acids and derivatives | Valine, leucine and isoleucine metabolism | HMDB  0000378 | N/A | 2-methylbutyroylcarnitine | 1.322 | 0.001 |
| PMID25887472 | Plasma | Organic acids and derivatives | Microbiome metabolism | HMDB  0000682 | N/A | Indoxyl sulfate | 1.263 | 0.001 |
| PMID25887472 | Plasma | Amino acids and derivatives | GABA, Glutamate, Arginine, Ornithine, Proline Metabolism | HMDB  0000267 | C01879 | Pyroglutamic acid | 1.379 | 0.01 |
| PMID25887472 | Plasma | Lipids | Lysophospholipids metabolism | HMDB  0010382 | N/A | LysoPC(16:0） | -0.786 | 0.03 |
| PMID25887472 | Plasma | Lipids | Lysophospholipids metabolism | HMDB  0010384 | C04230 | LysoPC(18:0) | -0.862 | 0.01 |
| PMID25887472 | Plasma | Lipids | Fatty Acid Oxidation and Synthesis | HMDB  0000736 | N/A | Isobutyrylcarnitine | 1.263 | 0.02 |
| PMID25887472 | Plasma | Lipids | Fatty Acid Oxidation and Synthesis | HMDB  0002366 | N/A | Tiglylcarnitine | 1.536 | 0.001 |
| PMID25887472 | Plasma | Lipids | Fatty Acid Oxidation and Synthesis | HMDB  0000201 | C02571 | Acetylcarnitine | 1.070 | 0.04 |
| PMID25887472 | Plasma | Lipids | Fatty Acid Oxidation and Synthesis | HMDB  0002013 | C02862 | Butyrylcarnitine | 1.263 | 0.02 |
| PMID26847922 | Plasma | Lipids | Lysophospholipids metabolism | HMDB  0010382 | C04230 | LysoPC(16:0) | -1.374 | 0.001 |
| PMID26847922 | Plasma | Lipids | Lysophospholipids metabolism | HMDB  0010384 | C04230 | LysoPC(18:0) | -0.927 | 0.003 |
| PMID26847922 | Plasma | Lipids | Lysophospholipids metabolism | HMDB  0010405 | C04230 | LysoPC(24:0) | -0.541 | 0.001 |
| PMID26847922 | Plasma | Lipids | Phospholipids metabolism | HMDB  0007875 | C00157 | PC(32:3) | -0.788 | 0.001 |
| PMID26847922 | Plasma | Lipids | Phospholipids metabolism | HMDB  0007883 | C00157 | PC(34:4) | -1.052 | 0.0001 |
| PMID26847922 | Plasma | Lipids | Phospholipids metabolism | HMDB  0007889 | C00157 | PC(36:4) | -0.735 | 0.0001 |
| PMID26847922 | Plasma | Lipids | Phospholipids metabolism | HMDB  0007881 | C00157 | PC(34:3) | -0.583 | 0.0001 |
| PMID26847922 | Plasma | Lipids | Phospholipids metabolism | HMDB  0007993 | C00157 | PC(40:1) | -0.952 | 0.002 |
| PMID26847922 | Plasma | Amino acids and derivatives | Tryptophan, Kynurenine, Serotonin, Melatonin Metabolism | HMDB  0000684 | C00328 | Kynurenine | 0.644 | 0.0001 |
| PMID27614981 | Serum | Lipids | Fatty Acid Oxidation and Synthesis | HMDB  0000201 | C02571 | Acetylcarnitine | -3.059 | 0.01 |
| PMID27614981 | Serum | Amino acids and derivatives | Urea Cycle | HMDB  0000904 | C00327 | Citrulline | -1.939 | 0.01 |
| PMID27614981 | Serum | Amino acids and derivatives | 1-Carbon, Folate, Formate, Glycine, Serine Metabolism | HMDB  0000043 | C00719 | Betaine | 1.678 | 0.01 |
| PMID27614981 | Serum | Amino acids and derivatives | Branch Chain Amino Acid Metabolism | HMDB  0000883 | C00183 | Valine | 1.585 | 0.01 |
| PMID27614981 | Serum | Amino acids and derivatives | Branch Chain Amino Acid Metabolism | HMDB  0000687 | C00123 | Leucine | 1.585 | 0.01 |
| PMID27614981 | Serum | Amino acids and derivatives | Branch Chain Amino Acid Metabolism | HMDB  0000172 | C00407 | Isoleucine | 2.644 | 0.01 |
| PMID27632672 | Plasma | Lipids | Eicosanoid and Resolvin Metabolism | HMDB  0001139 | C00639 | Prostaglandin F2alpha | 3.448 | 0.021 |
| PMID27632672 | Plasma | Lipids | Eicosanoid and Resolvin Metabolism | HMDB  0001085 | C02165 | Leukotriene B4 | 1.766 | 0.031 |
| PMID27632672 | Plasma | Lipids | Eicosanoid and Resolvin Metabolism | N/A | C18173 | Resolvin E2 | 1.319 | 0.019 |
| PMID27632672 | Plasma | Lipids | Eicosanoid and Resolvin Metabolism | HMDB  0004038 | N/A | Resolvin D5 | 4.087 | 0.022 |
| PMID27632672 | Plasma | Lipids | Eicosanoid and Resolvin Metabolism | N/A | N/A | 17R-protectin D1 | 2.585 | 0.035 |
| PMID27406941 | Plasma | Lipids | Lysophospholipids metabolism | HMDB  0011473 | N/A | LysoPE(16:0) | -1.434 | 0.002 |
| PMID27406941 | Plasma | Amino acids and derivatives | GABA, Glutamate, Arginine, Ornithine, Proline Metabolism | HMDB  0000267 | C01879 | Pyroglutamic acid | 0.422 | 0.007 |
| PMID27406941 | Plasma | Amino acids and derivatives | Tryptophan, Kynurenine, Serotonin, Melatonin Metabolism | HMDB  0000684 | C00328 | Kynurenine | 1.138 | 0.001 |
| PMID27406941 | Plasma | Amino acids and derivatives | Microbiome metabolism | HMDB  0000779 | C01479 | Phenyllactic acid | 0.888 | 0.001 |
| PMID27406941 | Plasma | Amino acids and derivatives | Fatty Acid Oxidation and Synthesis | N/A | N/A | N-6-trimethyllysine | 0.840 | 0.004 |
| PMID27406941 | Plasma | Nucleotides and derivatives | Polyamine metabolism and methionine salvage pathway | HMDB  0001173 | C00170 | 5-Methylthioadenosine | 0.642 | 0.018 |
| PMID30379669 | Plasma | Lipids | Fatty Acid Oxidation and Synthesis | HMDB  0000201 | C02571 | Acetylcarnitine | 0.441 | 0.018 |
| PMID25849571 | Serum | Amino acids and derivatives | Lysine Metabolism | HMDB  0000510 | C00956 | alpha-aminoadipic acid | 0.846 | 0.02 |
| PMID25849571 | Serum | Amino acids and derivatives | Tyrosine and Phenylalanine Metabolism | HMDB  0000159 | C00079 | Phenylalanine | 0.451 | 0.01 |
| PMID25849571 | Serum | Amino acids and derivatives | 1-Carbon, Folate, Formate, Glycine, Serine Metabolism | HMDB  0000187 | C00065 | Serine | -0.461 | 0.03 |
| PMID25849571 | Serum | Amino acids and derivatives | Taurine, Hypotaurine Metabolism | HMDB  0000251 | C00245 | Taurine | -0.752 | 0.01 |
| PMID12562829 | Plasma | Lipids | Sphingolipids metabolism | N/A | N/A | Total ceramides/total sphingomyleins | 0.737 | 0.001 |
| PMID12562829 | Plasma | Lipids | Lysophospholipids metabolism | N/A | N/A | Total LPC/PC | -0.857 | 0.001 |
| PMID28345042 | Plasma | Nucleotides and derivatives | Polyamine metabolism and methionine salvage pathway | HMDB  0001173 | C00170 | 5-Methylthioadenosine | 0.663 | 0.0024 |
| PMID31088568 | Serum | Amino acids and derivatives | Branch Chain Amino Acid Metabolism | HMDB  0000161 | C00041 | Alanine | 0.485 | 0.02 |
| PMID31088568 | Serum | Amino acids and derivatives | GABA, Glutamate, Arginine, Ornithine, Proline Metabolism | HMDB  0000148 | C00025 | Glutamic acid | -0.322 | 0.01 |
| PMID31088568 | Serum | Amino acids and derivatives | Bioamines and Neurotransmitter Metabolism | HMDB  0000641 | C00064 | Glutamine | 0.637 | 0.01 |
| PMID31088568 | Serum | Amino acids and derivatives | Tyrosine and Phenylalanine Metabolism | HMDB  0000159 | C00079 | Phenylalanine | 0.515 | 0.01 |
| PMID31088568 | Serum | Amino acids and derivatives | SAM, SAH, Methionine, Cysteine, Glutathione Metabolism | HMDB  0000562 | C00791 | Creatinine | 0.501 | 0.03 |
| PMID31088568 | Serum | Organic acids and derivatives | Glycolysis and Gluconeogenesis Metabolism | HMDB  0000190 | C00186 | Lactic acid | 0.515 | 0.03 |
| PMID31088568 | Serum | Organic acids and derivatives | Krebs cycle | HMDB  0000243 | C00022 | Pyruvic acid | 0.585 | 0.01 |
| PMID31088568 | Serum | Organic acids and derivatives | Krebs cycle | HMDB  0000094 | C00158 | Citric acid | 1.263 | 0.01 |

HMDB: Human Metabolome Database (http://www.hmdb.ca/); KEGG: Kyoto Encyclopedia of Genes and Genomes Database (<https://www.genome.jp/kegg/>).

# **Table S7 The vote count, pooled *P*-value, and Log2 fold change of the significantly altered metabolic pathways**

| **Metabolic pathways** | **Vote count** | **Pooled Log2 of fold change (Nonsurvivors/survivors)** | **95% CI for Log2 FC** | ***P*-value** |
| --- | --- | --- | --- | --- |
| Fatty Acid Oxidation and Synthesis | 15 | 0.72 | 0.64-0.81 | 0.014 |
| Branch Chain Amino Acid Metabolism | 14 | 0.49 | 0.42-0.56 | 0.024 |
| Lysophospholipids Metabolism | 11 | -0.93 | -0.99 - -0.86 | 0.006 |
| Amino-Sugar, Galactose, & Non-Glucose Metabolism | 8 | 0.94 | 0.84-1.01 | 0.011 |
| Glycolysis and Gluconeogenesis Metabolism | 6 | 0.42 | 0.34-0.51 | 0.017 |
| Phospholipids metabolism | 6 | -0.65 | -0.74 - -0.56 | 0.007 |
| SAM, SAH, Methionine, Cysteine, Glutathione Metabolism | 6 | 0.32 | 0.23-0.41 | 0.010 |
| Eicosanoid and Resolvin Metabolism | 5 | 2.64 | 2.41-2.91 | 0.026 |
| GABA, Glutamate, Arginine, Ornithine, Proline Metabolism | 5 | 0.36 | 0.26-0.47 | 0.009 |
| Microbiome Metabolism | 5 | 0.55 | 0.45-0.66 | 0.002 |
| Tryptophan, Kynurenine, Serotonin, Melatonin Metabolism | 5 | 0.83 | 0.73-0.94 | 0.006 |
| 1-Carbon, Folate, Formate, Glycine, Serine Metabolism | 4 | 0.05 | 0.04-0.06 | 0.013 |
| Krebs cycle | 4 | 0.67 | 0.54-0.81 | 0.019 |
| Bile acid metabolism | 3 | 1.05 | 0.79-1.31 | 0.001 |
| Bioamines and Neurotransmitter Metabolism | 3 | -0.08 | -0.14 - -0.023 | 0.030 |
| Tyrosine and Phenylalanine Metabolism | 3 | 0.59 | 0.45-0.73 | 0.007 |
| Urea cycle | 3 | 0.07 | 0.045-0.093 | 0.006 |
| Gamma-Glutamyl and other Dipeptides | 2 | 1.44 | N/A | 0.001 |
| Polyamine metabolism and methionine salvage pathway | 2 | 0.65 | N/A | 0.010 |
| Glycine, serine and threonine metabolism | 1 | 1.07 | N/A | 0.002 |
| Lysine Metabolism | 1 | 0.85 | N/A | 0.020 |
| Nicotinate and nicotinamide metabolism | 1 | 1.48 | N/A | 0.008 |
| Purine metabolism | 1 | 0.31 | N/A | 0.002 |
| Pyrimidine metabolism | 1 | 0.60 | N/A | 0.003 |
| Sphingolipids metabolism | 1 | 0.74 | N/A | 0.001 |
| Steroid metabolism | 1 | 1.63 | N/A | 0.004 |
| Taurine, Hypotaurine Metabolism | 1 | -0.75 | N/A | 0.010 |
| Tyrosine metabolism | 1 | 1.09 | N/A | 0.000 |
| Valine, leucine and isoleucine metabolism | 1 | 1.32 | N/A | 0.001 |
| Vitamin B6 metabolism | 1 | 2.65 | N/A | 0.001 |
| Vitamin E metabolism | 1 | 1.19 | N/A | 0.002 |

The vote count indicated the frequency of a metabolic pathway being statistically between survivors and nonsurvivors. The metabolic pathway for each differential metabolite was assigned based on HMDB and our in-house database. The combined fold change of each metabolic pathway was calculated by the meta-analysis using random-effects. 95% confidence interval (95% CI) was also provided. For the metabolic pathways with the vote count < 3, the average fold change was used. 95% CI was not calculated due to insufficient statistical power.

# **Table S8 Studies containing the direct comparisons of prediction accuracy for sepsis outcomes between metabolomics and traditional scores**

| Pubmed ID | Metabolites | AUC | SOFA AUC | APACHE AUC | SAPS II AUC |
| --- | --- | --- | --- | --- | --- |
| PMID24368342 | Thirty metabolites | 0.99 | 0.75 | 0.5 | N/A |
| PMID25928796 | Three metabolites | 0.99 | 0.81 | 0.78 | N/A |
| PMID31088568 | Alanine | 0.78 | 0.6 | N/A | N/A |
| PMID31088568 | Glutamate | 0.61 | 0.6 | N/A | N/A |
| PMID31088568 | Glutamine | 0.8 | 0.6 | N/A | N/A |
| PMID31088568 | Phenylalanine | 0.84 | 0.6 | N/A | N/A |
| PMID31088568 | Creatinine | 0.79 | 0.6 | N/A | N/A |
| PMID31088568 | Lactic acid | 0.74 | 0.6 | N/A | N/A |
| PMID31088568 | Pyruvic acid | 0.81 | 0.6 | N/A | N/A |
| PMID31088568 | Citric acid | 0.82 | 0.6 | N/A | N/A |
| PMID25849571 | Taurine | 0.67 | 0.86 | 0.86 | N/A |
| PMID28345042 | 5-Methylthioadenosine | 0.69 | N/A | 0.81 | N/A |
| PMID28345042 | 5-Methylthioadenosine | 0.79 | N/A | 0.76 | N/A |
| PMID12562829 | Total LPC/PC | 0.78 | N/A | N/A | 0.80 |

# **Table S9 Prediction accuracy of selected 7-analyte from DRMPs for sepsis death**

| Classifiers | AUROC (95% CI) | rdCV accuracy | Permutation P-value | Sensitivity (%)  (95% CI) | Specificity (%)  (95% CI) | Accuracy (%) | Positive Likelihood Ratio | Negative Likelihood Ratio | Diagnostic odds ratio |
| --- | --- | --- | --- | --- | --- | --- | --- | --- | --- |
| 7-analyte | 0.88 (0.78 - 0.97) | 0.773 | 0.002 | 80.4 (66.9 - 89.4) | 78.8 (62.3 - 89.3) | 80.1 (69.2 - 88.0) | 3.79 (1.93 - 7.43) | 0.25 (0.13 - 0.46) | 15.2 (5.04 - 46.2) |

Seven selected metabolites were: isoleucine (amino acid), alanine (amino acid), acetylcarnitine (mitochondrial metabolism), lactic acid (mitochondrial metabolism), pyruvic acid (mitochondrial metabolism), lysoPG (22:0) (lysophospholipids metabolism) and lysoPC (24:0) (lysophospholipids metabolism).


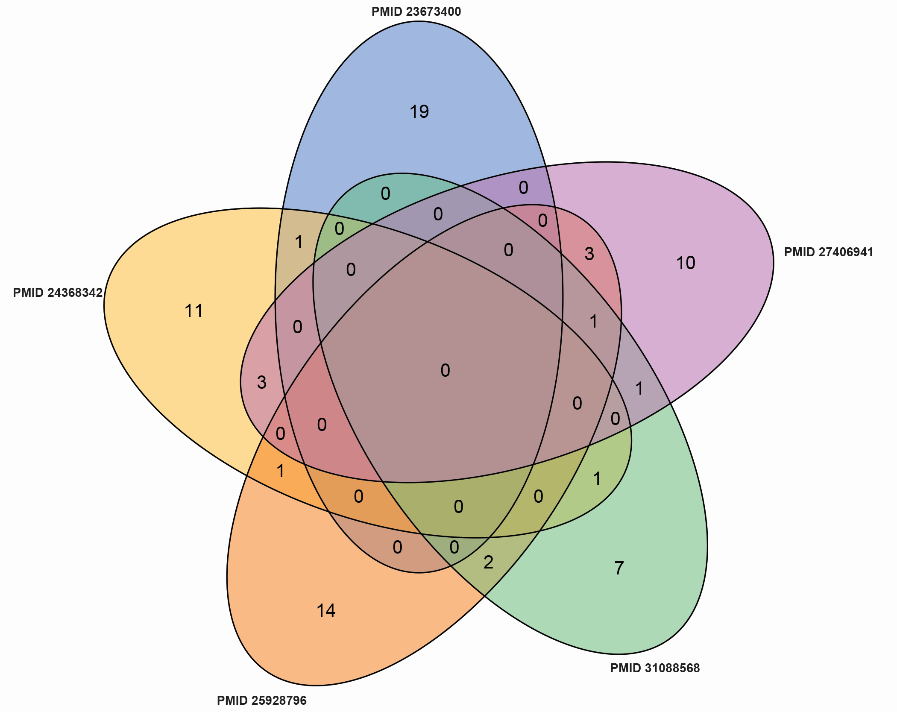


**Figure S1 Venn diagram showing the poor overlap of reported metabolite biomarkers across the studies for sepsis mortality prediction**


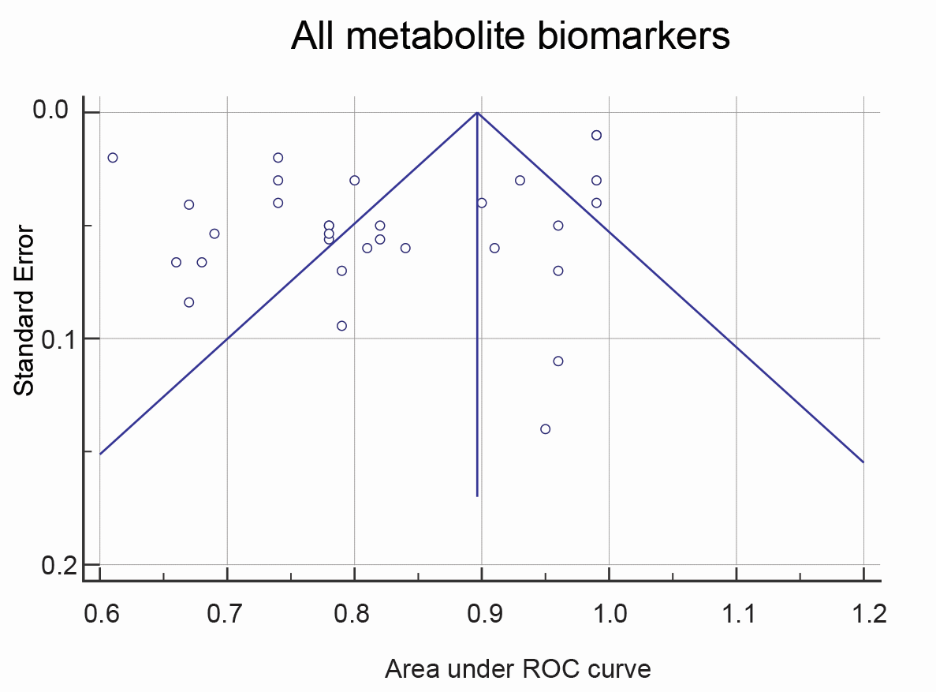


**Figure S2 The visualization of publication bias by the funnel plot for studies using metabolite biomarkers**


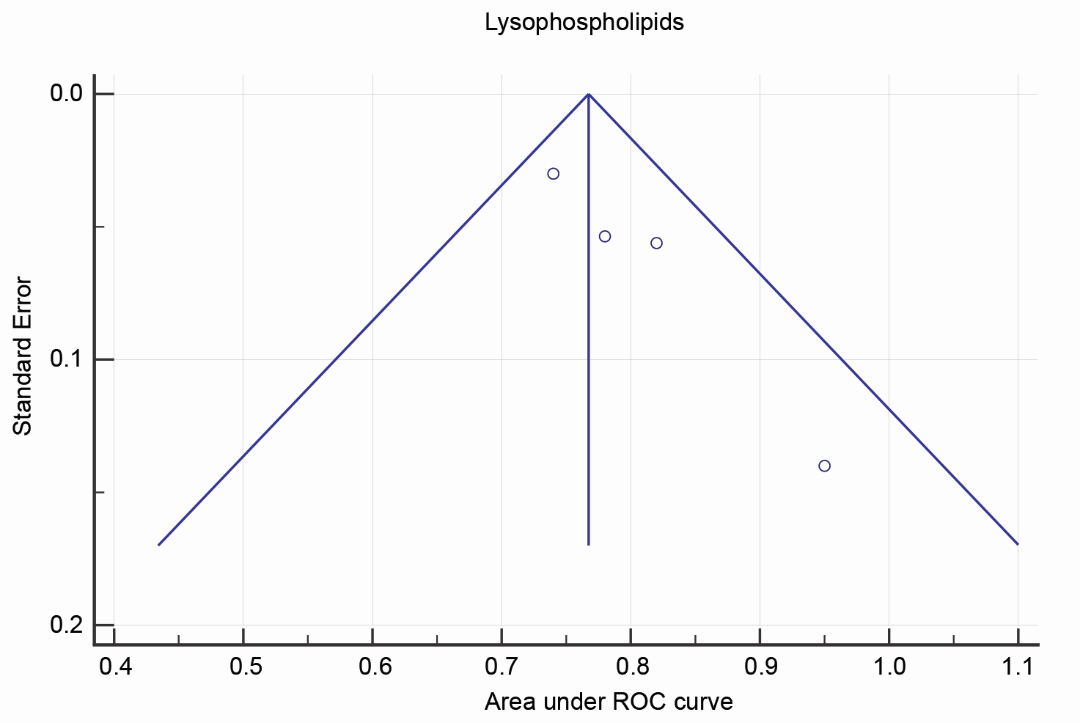


**Figure S3** **Assessment of publication bias by funnel plot for studies using lysophospholipids as biomarkers for sepsis outcome prediction**

**
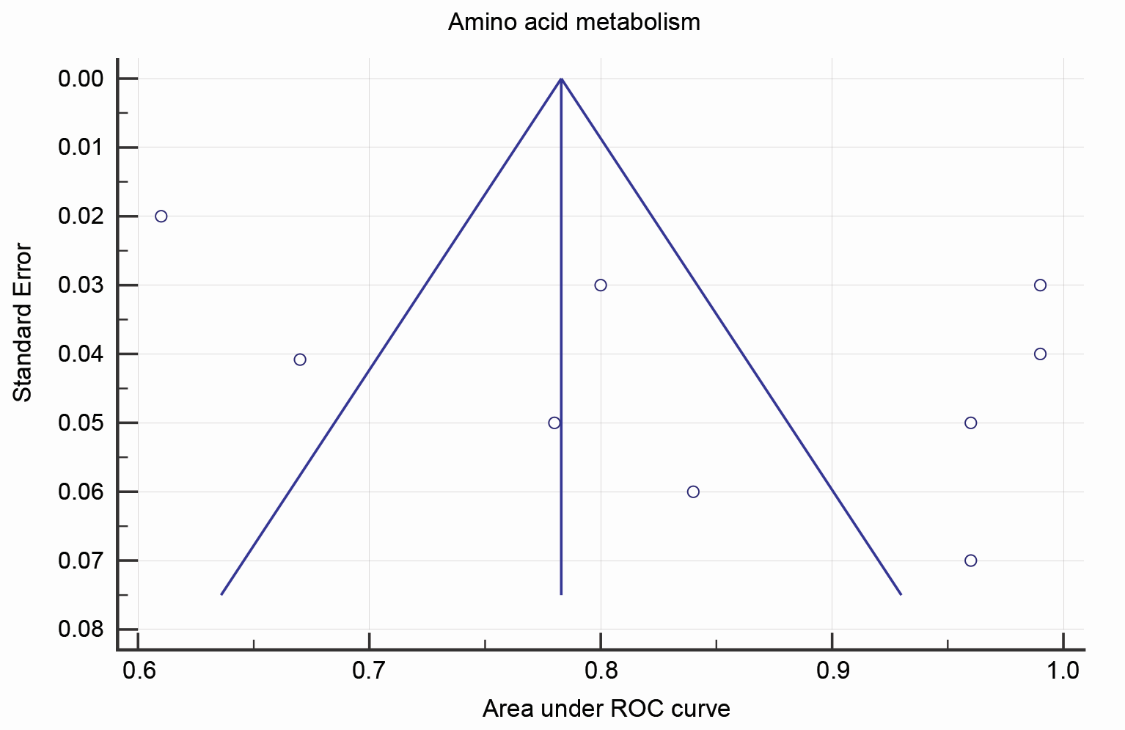
**

**Figure S4 Evaluation of publication bias by funnel plot for studies using amino acids as biomarkers for sepsis outcome prediction**

**
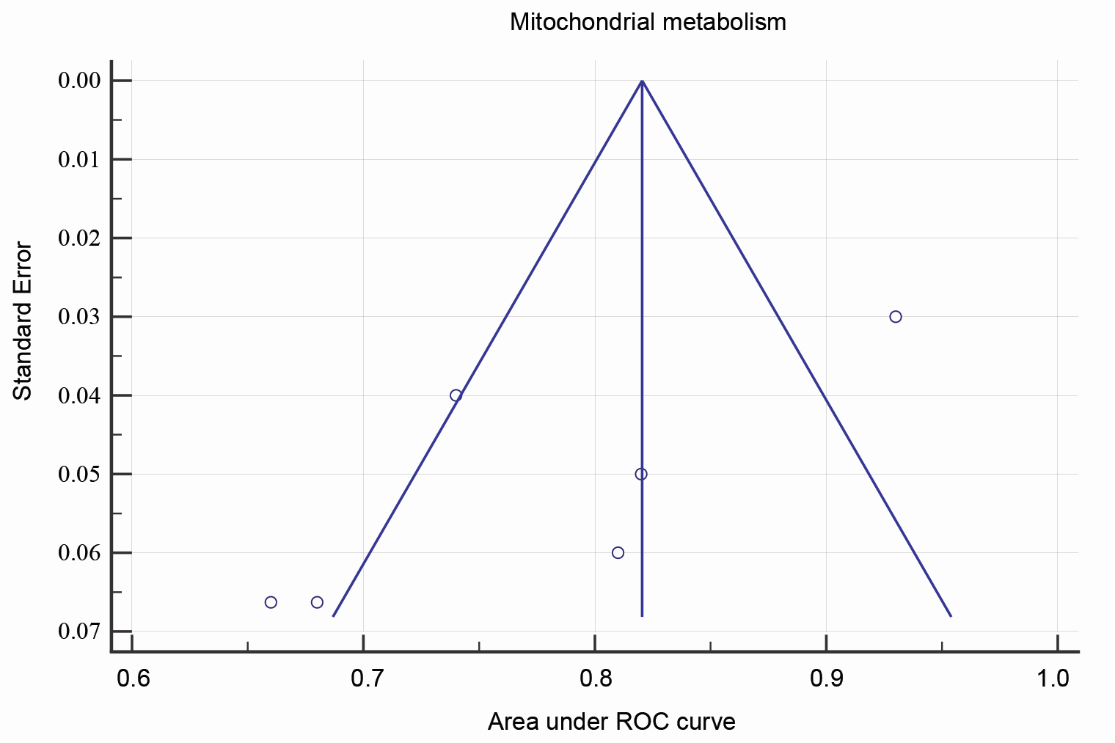
**

**Figure S5 Assessment of publication bias by funnel plot for studies using metabolites from mitochondrial metabolism as biomarkers for sepsis outcome prediction**

**
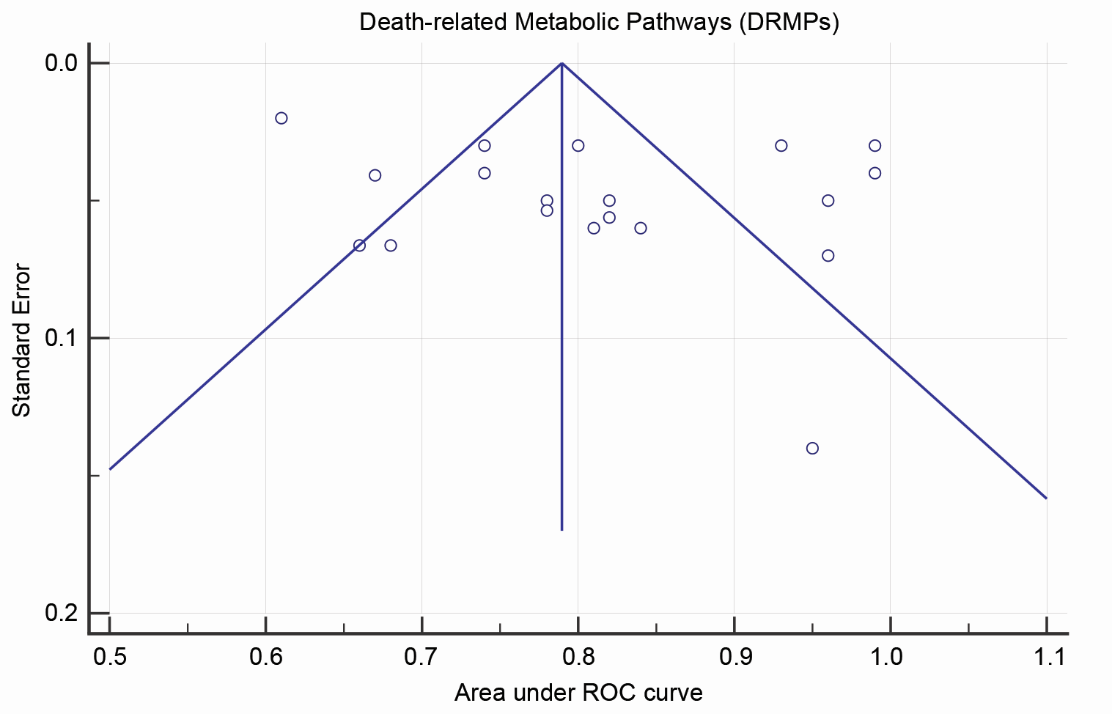
**

**Figure S6 Assessment of publication bias by funnel plot for studies using metabolites from DRMPs as biomarkers for sepsis outcome prediction**

**
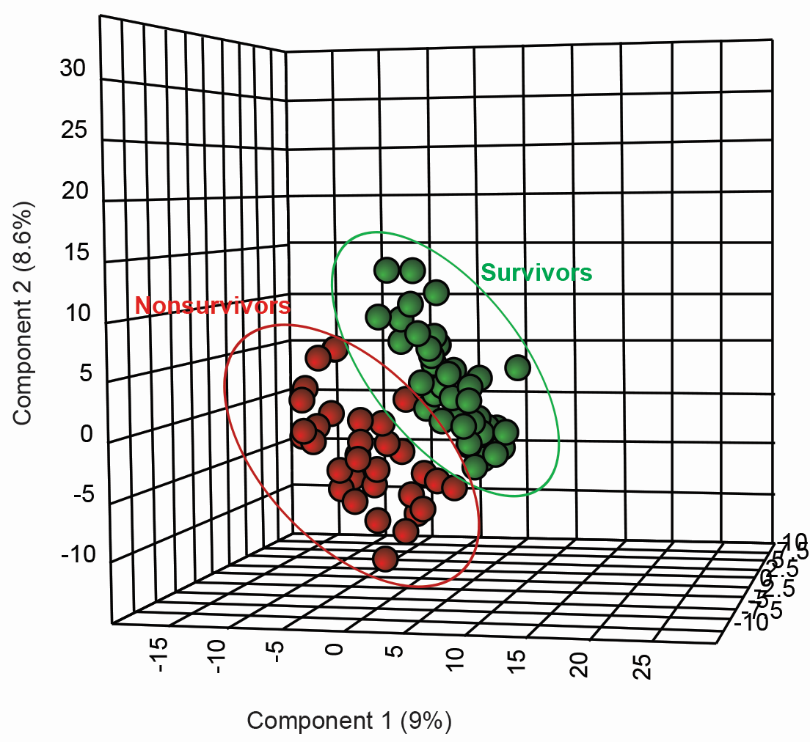
**

**Figure S7 3-D PLS-DA revealed the distinct separation of the plasma metabolome of sepsis nonsurvivors from sepsis survivors N = 134 for survivors and 54 for nonsurvivors.**

**
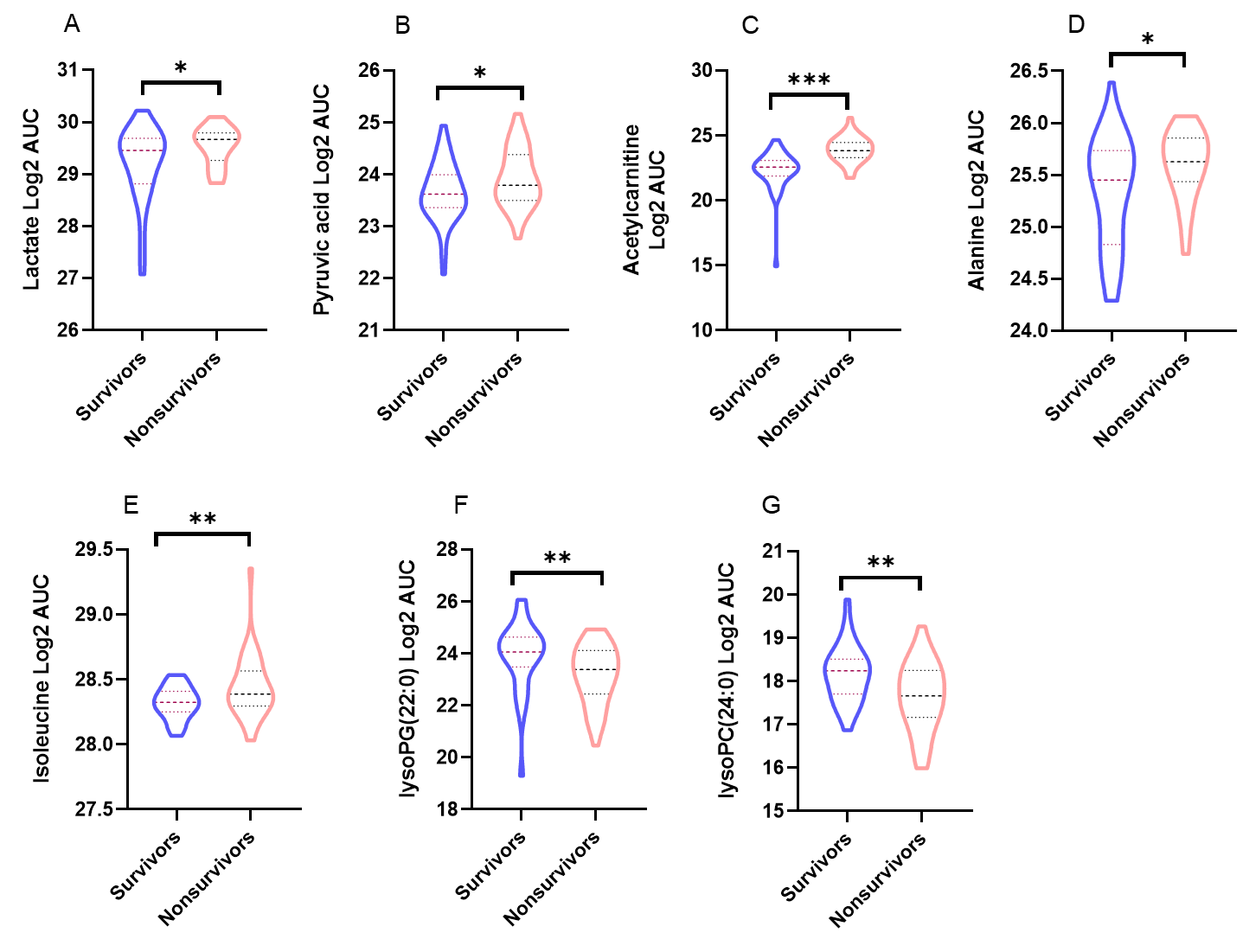
**

**Figure S8 The significant differences of 7 selected biomarkers from DRMPs between sepsis survivors and nonsurvivors**

Data were Log2 transformed and Mann-Whitney U test was performed. **P* < 0.05, ***P* < 0.01 and ****P* < 0.001. N = 134 for sepsis survivors and N = 54 for nonsurvivors. The median and quartiles were shown.


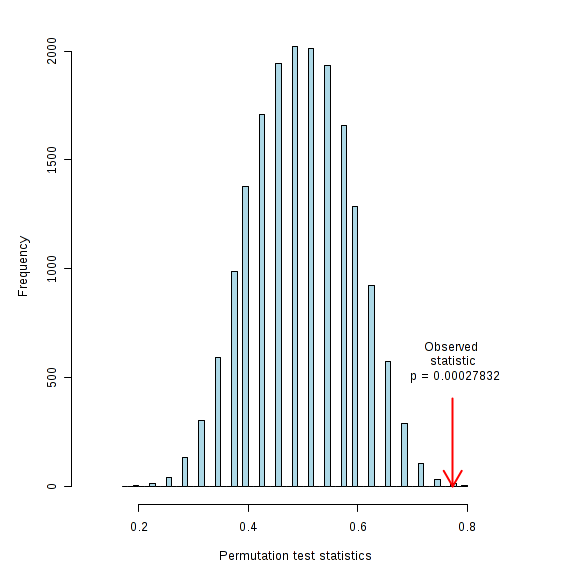


**Figure S9 The permutation test for the validation of the classification model robustness**

Permutation time: 500.

# **PRISMA checklist**

| **Section/Topic** | **#** | | | | **Checklist Item** | **Reported on Page #** |
| --- | --- | --- | --- | --- | --- | --- |
| **TITLE** | | | | | |  |
| Title | 1 | | | | Identify the report as a systematic review, meta-analysis, or both. | 1 |
| **ABSTRACT** | | | | | |  |
| Structured summary | 2 | | | | Provide a structured summary including, as applicable: background; objectives; data sources; study eligibility criteria, participants, and interventions; study appraisal and synthesis methods; results; limitations; conclusions and implications of key findings; systematic review registration number. | 2 |
| **INTRODUCTION** | | | | | |  |
| Rationale | 3 | | | | Describe the rationale for the review in the context of what is already known. | 3 |
| Objectives | 4 | | | | Provide an explicit statement of questions being addressed with reference to participants, interventions, comparisons, outcomes, and study design (PICOS). | 4 |
| **METHODS** | | | | | |  |
| Protocol and registration | 5 | | | | Indicate if a review protocol exists, if and where it can be accessed (e.g., Web address), and, if available, provide registration information including registration number. | 5 |
| Eligibility criteria | 6 | | | | Specify study characteristics (e.g., PICOS, length of follow-up) and report characteristics (e.g., years considered, language, publication status) used as criteria for eligibility, giving rationale. | 5 |
| Information sources | 7 | | | | Describe all information sources (e.g., databases with dates of coverage, contact with study authors to identify additional studies) in the search and date last searched. | 5 |
| Search | 8 | | | | Present full electronic search strategy for at least one database, including any limits used, such that it could be repeated. | 5 |
| Study selection | 9 | | | | State the process for selecting studies (i.e., screening, eligibility, included in systematic review, and, if applicable, included in the meta-analysis). | 5 |
| Data collection process | 10 | | | | Describe method of data extraction from reports (e.g., piloted forms, independently, in duplicate) and any processes for obtaining and confirming data from investigators. | 6 |
| Data items | 11 | | | | List and define all variables for which data were sought (e.g., PICOS, funding sources) and any assumptions and simplifications made. | 6 |
| Risk of bias in individual studies | 12 | | | | Describe methods used for assessing risk of bias of individual studies (including specification of whether this was done at the study or outcome level), and how this information is to be used in any data synthesis. | 6 |
| Summary measures | 13 | | | | State the principal summary measures (e.g., risk ratio, difference in means). | 6 |
| Synthesis of results | 14 | | | | Describe the methods of handling data and combining results of studies, if done, including measures of consistency (e.g., I^2^) for each meta-analysis. | 6 |
| Section/Topic | # | | | | Checklist Item | Reported on Page # |
| Risk of bias across studies | 15 | | | | Specify any assessment of risk of bias that may affect the cumulative evidence (e.g., publication bias, selective reporting within studies). | 6 |
| Additional analyses | 16 | | | | Describe methods of additional analyses (e.g., sensitivity or subgroup analyses, meta-regression), if done, indicating which were pre-specified. | 7 |
| **RESULTS** | | | | | | |
| Study selection | | 17 | | Give numbers of studies screened, assessed for eligibility, and included in the review, with reasons for exclusions at each stage, ideally with a flow diagram. | | 8 |
| Study characteristics | | 18 | For each study, present characteristics for which data were extracted (e.g., study size, PICOS, follow-up period) and provide the citations. | | | 9 |
| Risk of bias within studies | | 19 | Present data on risk of bias of each study and, if available, any outcome level assessment (see item 12). | | | 9 |
| Results of individual studies | | 20 | For all outcomes considered (benefits or harms), present, for each study: (a) simple summary data for each intervention group (b) effect estimates and confidence intervals, ideally with a forest plot. | | | 10 |
| Synthesis of results | | 21 | Present the main results of the review. If meta-analyses done, include for each, confidence intervals and measures of consistency. | | | 12 |
| Risk of bias across studies | | 22 | Present results of any assessment of risk of bias across studies (see Item 15). | | | 12 |
| Additional analysis | | 23 | Give results of additional analyses, if done (e.g., sensitivity or subgroup analyses, meta-regression [see Item 16]). | | | 13 |
| **DISCUSSION** | | | | | | |
| Summary of evidence | | 24 | Summarize the main findings including the strength of evidence for each main outcome; consider their relevance to key groups (e.g., healthcare providers, users, and policy makers). | | | 14 |
| Limitations | | 25 | Discuss limitations at study and outcome level (e.g., risk of bias), and at review-level (e.g., incomplete retrieval of identified research, reporting bias). | | | 17 |
| Conclusions | | 26 | Provide a general interpretation of the results in the context of other evidence, and implications for future research. | | | 18 |
| **FUNDING** | | | | | | |
| Funding | | 27 | Describe sources of funding for the systematic review and other support (e.g., supply of data); role of funders for the systematic review. | | | 20 |
